# Exploring the cognitive underpinnings of early hominin stone tool use through an experimental EEG approach

**DOI:** 10.1038/s41598-024-77452-0

**Published:** 2024-11-19

**Authors:** Simona Affinito, Brienna Eteson, Lourdes Tamayo Cáceres, Elena Theresa Moos, Fotios Alexandros Karakostis

**Affiliations:** 1 DFG Center for Advanced Studies “Words, Bones, Genes, Tools”, Department of Geosciences, Eberhard Karls University of Tübingen, Tübingen, Germany; 2 Paleoanthropology, Senckenberg Centre for Human Evolution and Palaeoenvironment, Department of Geosciences, Eberhard Karls University of Tübingen, Tübingen, Germany; 3 Integrative Prehistory and Archaeological Science, University of Basel, Basel, Switzerland

**Keywords:** Stone tool use, Electroencephalography, Human cognition, Brain evolution, Precision grip, Frontoparietal cortex, Biological anthropology, Electroencephalography - EEG, Archaeology

## Abstract

Technological innovation has been crucial in the evolution of our lineage, with tool use and production linked to complex cognitive processes. While previous research has examined the cognitive demands of early stone toolmaking, the neurocognitive aspects of early hominin tool use remain largely underexplored. This study relies on electroencephalography to investigate brain activation patterns associated with two distinct early hominin tool-using behaviors: forceful hammerstone percussion, practiced by both humans and non-human primates and linked to the earliest proposed stone tool industries, and precise flake cutting, an exclusive hominin behavior typically associated with the Oldowan. Our results show increased engagement of the frontoparietal regions during both tasks. Furthermore, we observed significantly increased beta power in the frontal and centroparietal areas when manipulating a cutting flake compared to a hammerstone, and increased beta activity over contralateral frontal areas during the aiming (planning) stage of the tool-using process. This original empirical evidence suggests that certain fundamental brain changes during early hominin evolution may be linked to precise stone tool use. These results offer new insights into the complex interplay between technology and human brain evolution and encourage further research on the neurocognitive underpinnings of hominin tool use.

**Supplementary Information:**

The online version contains supplementary material available at 10.1038/s41598-024-77452-0.

## Introduction

Tools have been an integral part of human evolution, shaping our ability to interact with and manipulate the world around us. Their significance extends beyond mere sustenance, as we have demonstrated a remarkable capacity to employ them skillfully and purposefully, enhancing their utility across diverse aspects of life. Technological advancement appears to align with the encephalization trend and modifications in brain structure that we observe within the genus *Homo*^1^, suggesting a correlation between them, albeit challenging to ascertain directly from the fossil record. Thus, comprehending the anatomical and cognitive demands associated with this technological revolution is fundamental for understanding the trajectory that enabled us to employ tools in the sophisticated manner seen today.

The emergence of the first lithic technology and its implications for early hominin cognition and behavior have been central topics in evolutionary science. Traditionally, the Oldowan industry, dating back to ~ 2.6 million years ago^2–4^, characterized by sharp-edged flakes, has been considered the earliest stone tool industry, hypothesized to reflect the emergence of habitual human-like precision grasping, skilled object manipulation, and cognitive complexity^5–8^. However, the idea that tool use abilities are exclusive to our genus has undergone substantial reconsideration over the years. In addition to recent findings suggesting that the Oldowan industry had a wider distribution earlier than previously thought^9^, the production of stone artifacts can currently be traced back to ~ 3.3 million years ago, with the emergence of the Lomekwian industry predating the genus *Homo*^10^ (but see^11,12^ for the debate surrounding the Pliocene origin of this lithic assemblage). Moreover, several animal species, including wild communities of great apes and monkeys, exhibit a variety of tool using behaviors (e.g.,^13–15^).

Recent research has documented macaque and capuchin monkeys, while engaged in pounding activities, repeatedly and unintentionally producing conchoidal flakes that fall in the variability range of the early Oldowan and Lomekwian tool assemblages^16,17^. This discovery prompts a cautious reevaluation of assumptions regarding the intentional manufacture of early stone tools. It suggests that, excluding deliberate tool-making intentions such as raw material selection, there may exist similarities between the percussive behaviors of early hominins and those observed in non-human primates. The Lomekwian artifacts, for instance, are proposed to have been produced using passive hammer and bipolar techniques, echoing the tool use pounding actions observed in primate behaviors^10^. This correlation aligns with the hypothesis that the earliest cutting tools could have emerged as byproducts of percussive activities, such as nut-cracking, thereby serving as precursors to deliberate stone tool knapping^18–20^.

Although the exact timing and circumstances of intentional tool production require further investigation, the introduction of cutting tools represents a significant evolutionary transition, also evidenced by some of the earliest direct indications of butchery marks dating back to approximately 2.6 million years (e.g.,^21,22^; but see^23^ for the controversial case from Dikika, and^24^ for discussion about fundamental shifts and continuity in hominin behavior). In contrast to percussive activities shared with non-human primates, the habitual use of sharp-edged tools for cutting appears to be a distinctive hominin behavior. This distinction suggests a pivotal moment in our evolutionary framework where the adoption of cutting tools likely provided adaptive advantages to our ancestors and influenced their subsistence strategies^25,26^.

While the integration of brain imaging techniques with behavioral and archaeological data has enriched our understanding of the neurocognitive aspects involved in intentional early stone tool-making and the shared network between speech and manual praxis (e.g., see^27–29^), the cognitive aspects of early stone tool use have been comparatively overlooked. This could be attributed to the reasonable emphasis placed on shifts in the complexity and sophistication of stone tool production, alongside the growing evidence of tool use across various animal species, contributing to identify production rather than use as a defining feature that sets humans apart from other species, particularly non-human primates (but see discussion above on intentionality and early stone tool industries). However, there are differences between human and non-human primates regarding tool brain functions, such as in action control, visuomotor coordination, and mechanical knowledge^30–32^. Furthermore, a pre-existing comprehension of the function and utility of tools is likely to underlie the acquisition of skills, such as understanding fracture mechanics and sensorimotor control^33^, which are essential for the intentional production of tools.

Our current understanding of the neurocognitive underpinnings of human tool use relies on functional neuroimaging studies conducted primarily on left brain-damaged patients. The neural network implicated in human tool use typically involves specialized regions within the left parietal, frontal, and temporal cortices, which coincide with brain areas that have been suggested to have undergone evolutionary changes (e.g.,^34^). Two studies^35,36^ provide a comprehensive and critical overview of these neuroimaging findings. As a general outline, the more dorsal regions of the parietal lobe are primarily involved in sensorimotor control, such as visuospatial integration^37,38^. In contrast, more ventral areas, particularly in the left inferior parietal lobe, play a crucial role in understanding mechanical actions or mechanical knowledge^39,40^ rather than directly controlling motor actions. The role of frontal regions in tool use remains less clearly defined. More ventral areas of the prefrontal cortex have been associated with higher-level executive functions^41^ and may be key in integrating diverse forms of action-related information^42^. Meanwhile, the temporal cortex is critical for tool-related semantic processes, serving as a hub for the multimodal integration of semantic knowledge^42,43^.

These studies have predominantly employed functional magnetic resonance imaging (fMRI), renowned for its exceptional spatial resolution, allowing precise brain activation mapping. However, the temporal dynamics might be overlooked. For instance, the use of tools involves a sequence of cognitive and motor functions. It requires the ability to associate the physical properties of the tool with the characteristics of the object and understand how the tool can be used effectively to interact with the object in order to achieve the intended outcome (e.g., see^32,36^). This selection process is guided by mechanical knowledge, as highlighted by studies on tool-use disorders in left parietal brain-damaged patients, where impairments in tool selection hinder tool-use ability (e.g.,^40,44^). As the tool is functionally grasped, it is directed toward the object of the intended action, and then the action is executed to achieve the goal. These individual motor components have rarely been investigated in a unified real-time experimental setting using a high-time resolution approach, such as electroencephalography (EEG). This technique has been successfully used in motion-related studies (e.g.,^45^).

This study aims to explore the cognitive requirements of early hominin stone tool use within a real-time experimental framework, allowing the comparison of cognitive processes across different stages of tool use. Specifically, our focus is on two of the earliest documented patterns of stone tool use: nut-cracking, generally associated with power grasping, and flake cutting, generally associated with precision grasping. While nut-cracking behaviors are observed in other primates, the intentional use of cutting tools is typically attributed to hominins^46,47^. Our objective is to ascertain whether flake cutting elicits a greater involvement of frontal-parietal areas, which are crucial for human-like tool manipulation and which have been subjected to substantial evolutionary pressures. Building on existing literature^31,35,48^, we hypothesize that both patterns of stone tool use will prominently involve fronto-parietal regions (Hypothesis 1). However, we also expect that precise cutting will show a greater engagement in these areas compared to nut-cracking (Hypothesis 2), reflecting these regions’ central role in human brain evolution and the proposed exclusive association between cutting and hominin contexts. Furthermore, we predict that the planning stage of stone tool use actions will exhibit the strongest signal among all stages (Hypothesis 3), in line with neurological literature on human tool use^49,50^.

## Methods

### Subjects

A total of twenty-five participants were recruited for this experiment, primarily from the University of Tübingen, and included individuals from 10 different nationalities and various professional backgrounds. In this pool, 23 individuals were self-identified as right-handed. The two left-handed individuals (two biological males) were not included in this study to ensure a more homogeneous sample in terms of handedness and brain lateralization patterns. Our final sample consists of 14 biological females and 9 biological males aged 22–55 years (mean age = 30.7 ± 7.4 years). Participants had no previous history of neurological and psychiatric disorders, as well as hand pathologies that could potentially affect task execution. Each subject signed an informed consent document prior to the experiment. This study was formally approved by the Ethics Committee for Psychological Research of the University of Tübingen, in line with the international recommendations of the Declaration of Helsinki (1964, revised in 2013).

## Stone tools and materials

Forty stone-tool replicas (17 quartzite hammerstones and 23 flint flakes) were used in this study (Supplementary Figure S1). All replicas were sourced and knapped by one of the authors (E.M. – an expert toolmaker with 10 years of experience) to mirror the size and shape observed in the archaeological record. The hammerstones ranged from 8 to 14 cm in length^51^, while the flakes measured between 5 and 7 cm, in conformity with documented proportions of Oldowan flakes^52,53^. Participants were given the chance to select the tool they perceived as more comfortable and proportionate to their hand sizes, aiming to facilitate their engagement in the assigned task.

For the nut-cracking task, we used macadamia nuts (*Macadamia* *integrifolia*), which were purchased from TALI e.K. (Helsa, Germany). These have been employed in previous studies (e.g.,^20,54,55^), due to their ready availability and the comparable hardness of their shells to those (e.g., *Elaeis guineensis* and *Panda oleosa*) documented being cracked open by wild chimpanzee communities in West Africa^56–58^. Before being used in our experiment, macadamia nuts were lightly roasted due to their excessively hard shells (making them almost impossible to crack), using the same heating equipment and temperature for all of them. This strategy was followed because, during our trial experiments, participants faced difficulty in opening the nuts, leading to frustration and intensified efforts to crack them (unsuccessfully), therefore impacting the quality of our EEG recordings. Light roasting rendered the nuts breakable, albeit still considerably resistant.

**Fig. 1:**
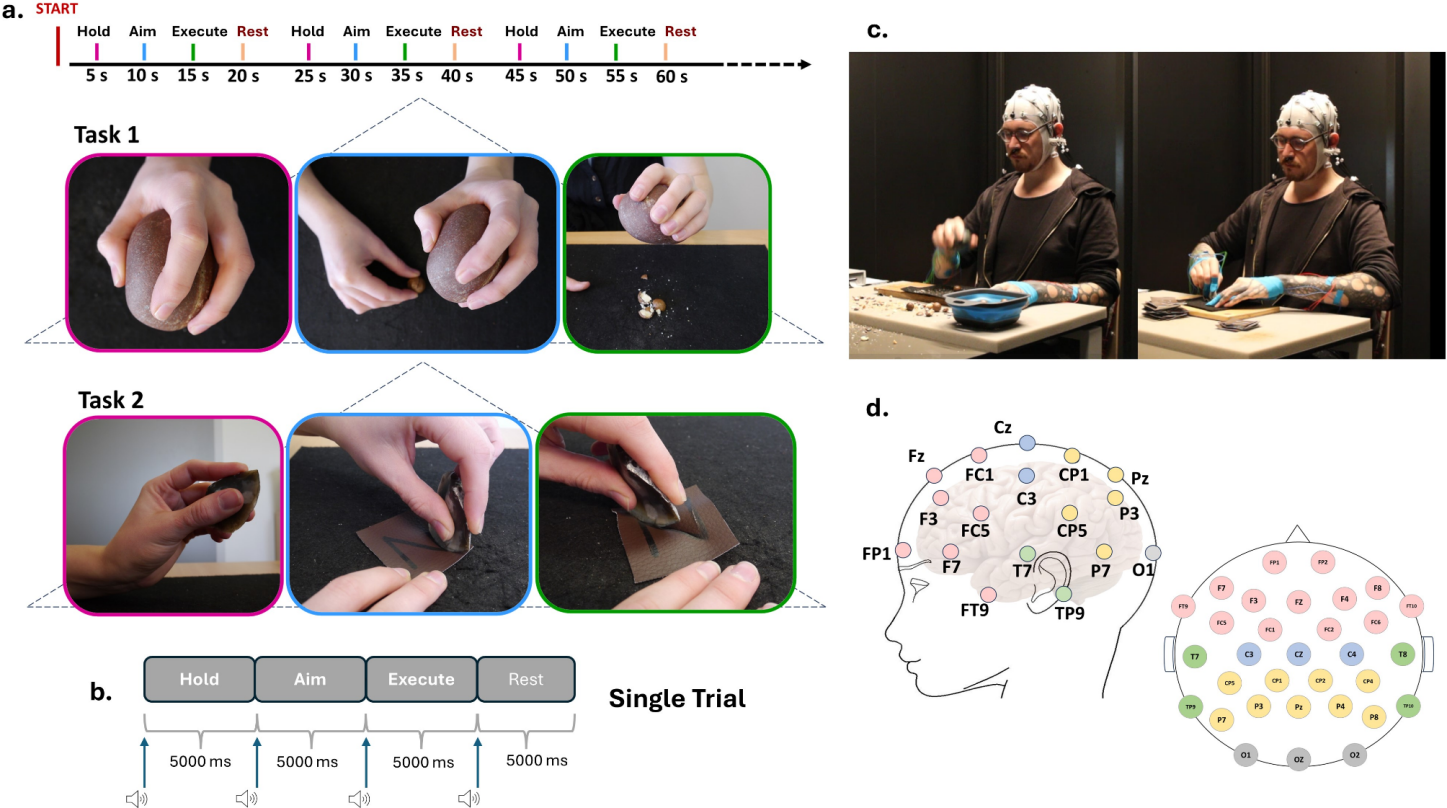
Experimental setup. (**a**) Schematic representation of the nut-cracking (Task 1) and cutting (Task 2) tasks. The sequence of images represents the “Hold” (pink frame), “Aim” (blue frame), and “Execute” (green frame) steps. (**b**) Single trial with a duration of 20 s. The arrows represent the beeping sound marking the beginning of each step. (**c**) Experimental environment and an example demonstrating the cracking (left) and cutting (right) steps. This is not a picture taken during the experiments of this study. (**d**) Location of the 32 electrodes (pink = frontal cortex; blue = motor cortex; green = temporal cortex; yellow = parietal cortex; grey = occipital cortex) from which EEG signals were recorded. The electrodes are placed according to the international 10–20 system.

For the cutting task, we used squares made of faux leather. We opted for faux leather, instead of real leather, primarily to ensure consistency across all trials by eliminating the variability often present in natural materials (e.g., texture, thickness). To ensure uniformity in participant performance during the cutting task, a distinctive Z shape (a pattern of 3 interconnected lines, each measuring 3 cm) was drawn on every square (see Fig. 1). Although the immediate objective of this task may not perfectly replicate early stone-tool cutting activities, a similar approach has already been employed in previous studies involving Palaeolithic cutting tools^59^, and it guarantees a standardized engagement in a dynamic and precise motor control task.

## Experimental design

The experiment consisted of two stone-tool-using tasks: a nut-cracking task (Task 1), where participants used a hammerstone replica to crack a macadamia nut; and a cutting task (Task 2), where participants employed an Oldowan flake replica to cut a pre-marked ‘Z’ outline through a faux leather square. In each task, participants were instructed to perform the action in three distinct and consecutive steps: “Hold”, “Aim”, and “Execute” (Fig. 1a).

In the “Hold” step, participants had to pick up the tool with their dominant hand, employing a power grip for the hammerstone and a precision grip for the flake. No specific indications were provided to the participants regarding the type of grip. Consequently, all participants naturally adopted a spherical five-jaw power grip and, for the precision grip, either a two-jaw chuck pad-to-side or a three-jaw chuck pad-to-side with the second digit used in forceful opposition to the cutting edge (^60^or see^61^ for a description of these two grip types). In the “Aim” step, participants were required to prepare for the execution by aiming at the target. To accomplish this, they were instructed to use their non-dominant hand to pick up either the nut or the faux leather square, place the materials on the platform, and then prepare to strike or cut by positioning the tool in the direction of the target. In the “Execute” step, participants finally cracked the nut or executed the cutting action. During the flake cut, all subjects used the non-dominant hand to steady the faux leather square.

Through the deliberate division of the task into these 3 steps, we wanted to isolate and explore the different components inherent in using a tool (e.g., functional grasping, planning, and executing tool use actions). Participants were given a time of 5 s to perform each step, prompted by an auditory cue (duration 30 ms) signaling the start of the subsequent action. Following the completion of the three steps, an additional 5-second resting period was incorporated to reset and clear the platform before starting the next repetition. As depicted in Fig. 1b, the overall trial duration lasted 20 s. Upon achieving a minimum of 50 trials, participants were granted a 30-minute break before progressing to the next task.

Prior to the experiment, participants were provided with an instructional video, offering a thorough description of the experimental tasks and the EEG application. This information was intended to guarantee that participants were well-prepared and had a clear understanding of the upcoming experiment. Alongside this, they received a set of guidelines to follow in preparation for their planned sessions, including instructions to abstain from consuming alcohol and caffeine for 12 h.

The EEG recording sessions were conducted within a shielded cabin to minimize any electromagnetic interference, at the Max Planck Institute for Intelligent Systems, in Tübingen (see Acknowledgments). Within this cabin, participants were seated comfortably in a chair positioned in front of a table equipped with a striking platform (a wooden cutting board on a thick polyethylene foam sheet), the tool, and either a basket with nuts or a pile of faux leather squares (Fig. 1c – informed consent was obtained to publish this image in an online open access publication). At the start of each experimental session, participants were asked to perform a simple action of closing and opening their right hand without manipulating any tools. This action was recorded as a control task (meaningless movement). Following this, they were given a few minutes to practice the nut-cracking and cutting tasks. Participants were directed to focus on each step by maintaining the ongoing action until the next auditory cue, and to uphold a proper posture by keeping their back against the chair. Additionally, we asked to minimize facial expressions as well as head and body movements, to reduce muscular artifacts.

## EEG data acquisition and preprocessing

EEG data were acquired from 32 electrodes mounted on an elastic cap and positioned according to the International 10–20 system (Fig. 1d) using a BrainAmp amplifier and BrainVision Recorder software (Version 1.24.0101, Brain Products GmbH, Gilching, Germany). The central frontal electrode FCz was used as the online reference, and the EEG signal was recorded at a sampling rate of 2500 Hz. This high sampling rate was selected due to the simultaneous recording of electromyographic (EMG) data, although the results of the EMG analysis are not presented here. The electrodes were filled with an electrode gel to improve connection and achieve a low impedance (below 20 kΩ). In the continuous EEG recording, four markers were manually introduced to indicate the three distinct steps and the resting period. Furthermore, as mentioned in the previous paragraph, an auditory cue was played every five seconds. This sound was recorded simultaneously in the software, enabling us to associate each beep with the beginning of the corresponding step and make adjustments during data preprocessing.

EEG data were preprocessed in BrainVision Analyzer (Version 2.2, Brain Products GmbH, Gilching, Germany). Initially, the raw data were visually inspected, and noisy or dead channels were identified and removed. Since markers were manually introduced in the continuous EEG recording, their positions were adjusted according to the beeping sound signal. During the experiment, a small window in the shielded cabin facilitated real-time observation of participants, enabling the monitoring and documentation of participant performance. Therefore, we were able to exclude incorrectly executed trials by removing the corresponding markers. Then, data were downsampled to 250 Hz, filtered using an IIR filter with a low cutoff set at 1 Hz and a high cutoff at 40 Hz, and notch-filtered at 50 Hz to remove power line disturbances. Filtered data were re-referenced using the Reference Electrode Standardization Technique^62^ (REST). In order to remove ocular artifacts, an automatic Independent Component Analysis (ICA) was applied using the Infomax algorithm^63,64^. The components relative to eye blinks and eye movements were marked, and the remaining were back-projected onto the channels using the Inverse ICA transformation. Components that displayed a suspicious topography, indicating, for example, persistent muscular artifacts during the recording, were also removed (for some examples of topography, see Fig. 1 from^65^). Before deselecting these components, we visually assessed the impact of this removal on the data to avoid overcorrection. On average, seven components were deselected across all participants. Bad channels and\or previously removed channels, were then subjected to interpolation.

Segments of 15,200 ms were created through an initial segmentation. These include a baseline period at -200 ms and the entire trial (all three steps), excluding only the resting period. The segments were then baseline corrected. This approach was adopted to prevent the potential inclusion of task-related activity in the baseline. As previously mentioned, the three experimental steps were executed in sequence. Therefore, if the above process had not been followed, for the Aim and Execute, the baseline would have been within the holding and aiming intervals, respectively.

Throughout this process, data inspection was done twice. Firstly, we manually inspected the data before Independent Component Analysis. This was done to mark large artifacts that we did not want to be computed by ICA. Secondly, we performed a semi-automatic inspection before segmentation to mark and visually re-check for bad intervals.

## EEG data analysis

We performed a conditional segmentation based on markers, with each step epoched from 0 to 1000 ms. Unfortunately, the hammerstone Execute step was removed from our analysis due to an artifact arising at around 400 ms, from the impact of the hammerstone with the nut. Despite our best efforts, we were unable to remove this artifact with the software we used without compromising the integrity of the data. Attempts to use a shorter segment (from 0 to 350 ms) proved unsuccessful. As a result, we have focused our analysis on a total of five steps, encompassing all three steps for Task 2 and the first two steps for Task 1.

Segments flagged for artifact contamination were rejected. To discern significant variations in beta frequency across different tasks and experimental steps, we performed a Fast Fourier Transformation (FFT) on each single trial. A Hanning window (10%) was applied to compute the power spectrum, and then we averaged across trials in order to compute the mean power spectrum for each task step per subject. The mean number of trials included in the averaging process was 44, in line with EEG study parameters. Subsequently, we extracted the mean beta activity (µV) within the 12.5–30 Hz frequency range for each participant. Topographic maps were generated using grand-averaged mean spectra (all topographic maps were produced in BrainVision Analyzer - Version 2.2, Brain Products GmbH, Gilching, Germany), while statistical analyses were conducted on individual mean power spectra.

All the following analyses were performed in R (4.1.0 version). To identify EEG channels showing significant differences in beta power across distinct steps and tasks, our analytical approach involved comparing two groups at a time: first, by examining the same condition (e.g., holding the tool) across different tasks, and second, by comparing each step within the same task. These analyses were executed using paired statistical tests, specifically employing the Wilcoxon signed-rank test, following a similar approach used by^66^. As a precaution, channels Fp1, Fp2, TP10, TP9, FT10, and FT9 were excluded from the statistical analysis since, during data preprocessing, we recognized these channels as potentially prone to noise (in line with usual practice in EEG). Based on the outcomes of the paired Wilcoxon test, we identified five channels where the differences were deemed significant (for each comparison; *p* < 0.05), organizing them in ascending order based on the magnitude of their Z scores (as a reflection of the effect size). In instances where we observed fewer than five channels with statistical significance, our criterion for selection involved choosing the top five channels with the highest absolute Z scores. This approach ensured a robust and prioritized selection of channels reflecting notable variations in beta power.

Furthermore, to evaluate whether a multivariate combination of these chosen channels (patterns of correlated channels) enables effective differentiation between tasks or steps, we conducted a Principal Component Analysis (PCA) for each paired comparison. These analyses were based on a correlation matrix, using the mean power values of the five selected channels as variables^67^. A scree-plot approach was used to determine the number of principal components (PCs) to plot. Additionally, due to high inter-individual variability combined with the fact that each individual was represented twice within each PCA plot (one for each step/task), the paired (intra-individual) trends between the two compared tasks were clearly present but required a certain effort to visually discern. Therefore, we facilitated the interpretability of our plots by subtracting, for each individual, the mean between the two tasks/steps from the power values of each task/step, an approach already used in previous studies on human bilateral asymmetry (e.g.,^68^). It is worth clarifying that this study’s reported patterns were still present in the PCAs before implementing this adjustment (e.g., see Supplementary Figure S2 with paired differences in PC scores). Finally, a Wilcoxon test was conducted on the extracted PC scores to assess the statistical significance (*p* < 0.05) of differences between the two groups (steps and/or tasks) on each PC axis. If outliers were detected in PC scores - determined using boxplots and the interquartile range approach - on the PC axis where statistically significant differences were found, the entire process (Wilcoxon tests and PCAs) was repeated without the outliers.

## Results

The results obtained from the paired comparisons are presented in the following section and outlined in Table 1. The first group of analyses encompasses comparisons examining the same condition across two distinct tasks (first two paragraphs). Meanwhile, the second group of analyses focuses on comparisons between steps within the same task (last paragraph). In the PCAs, emphasis was placed on the first two principal components (PC1 and PC2), which together reflect the main variation and significant differences.

**Table 1:** PCA-selected channels for paired comparisons.

Paired comparisons		Hold vs. Hold	Aim vs. Aim	Task 1 (hammerstone nut-cracking)			Task 2 (flake cutting)					
				Control vs. Hold	Control vs. Aim	Hold vs. Aim	Control Vs Hold	Control Vs Aim	Control Vs Cutting	Hold vs. Aim	Aim vs. Cutting	Hold Vs Cutting
**Channels (p-value/** **Z-score)**	Fz	-	-	-	-	**0.001/** **-3.2**	-	-	-	-	-	-
	F3	-	0.06/ -1.9	-	**0.0002/** **-3.8**	**0.0001/ -3.8**	**0.002/** **-3.2**	**9.78E-05/** **-4**	-	**0.001/ -3.3**	**9.91E-05**/ **-4**	-
	F7	-	-	-	-	-	-	-	-	-	-	-
	FC5	-	-	-	-	-	-	**0.003/** **-3**	-	**0.0004/ -3.5**	-	-
	FC1	**0.03/** **-2.1**	-	**0.02/** **-2.3**	**0.002/** **-3.2**	**0.002/ − 3.1**	**0.02/** **-2.4**	**0.001/** **-3.3**	-	-	**0.0003/** **-3.6**	-
	C3	-	-	-	**0.001/** **-3.3**	**0.01/** **-2.8**	-	**0.001/** **-3.5**	-	-	**0.001/** **-3.5**	-
	T7	-	-	-	-	-	-	-	-	**0.0002/ -3.7**	-	-
	CP5	-	-	-	-	**0.01/** **-2.8**	-	-	-	**0.002/** **-3.1**	-	-
	CP1	**0.03/** **-2.2**	-	-	-	-	**0.02/** **-2.4**	**0.001/** **-3.3**	-	-	-	-
	Pz	-	-	-	**0.01/** **-2.6**	-	-	-	-	-	-	**0.0003/** **-3.7**
	P3	-	-	-	-	-	-	-	-	-	-	-
	P7	-	-	-	-	-	-	-	-	-	-	-
	O1	-	-	-	-	-	-	-	0.09/ -1.7	-	-	-
	Oz	-	0.06/ -1.9	-	-	-	-	-	-	-	-	-
	O2	**0.02/** **-2.3**	**0.01/** **-2.8**	-	-	-	-	-	-	-	-	-
	P4	-	0.07/ -1.8	**0.002/** **-3.1**	-	-	**0.02/** **-2.4**	-	-	-	**0.0003/** **-3.6**	**0.0003/** **-3.6**
	P8	**0.02/** **-2.3**	**0.02/** **-2.4**	**0.003/** **-3**	**0.001/** **-3.2**	-	-	-	-	-	-	-
	CP6	-	-	**0.02/** **-2.4**	-	-	-	-	-	-	-	-
	CP2	-	-	-	-	-	-	-	-	-	**0.0002/** **-3.7**	**4.30E-05/** **-4**
	Cz	**0.04/** **-2.1**	-	-	-	-	**0.003/** **-3**	-	-	-	-	**0.0001/** **-3.9**
	C4	-	-	**0.001/** **-3.4**	-	-	-	-	**0.01/** **-2.6**	**0.0002/** **-3.7**	-	-
	T8	-	-	-	-	-	-	-	-	-	-	-
	FC6	-	-	-	-	-	-	-	0.07/ -1.8	-	-	-
	FC2	-	-	-	-	-	-	-	**0.02/** **-2.4**	-	-	**6.35E-05/** **-4**
	F4	-	-	-	-	-	-	-	0.1/ -1.5	-	-	-
	F8	-	-	-	-	-	-	-	-	-	-	-

This table presents the channels chosen for principal component analysis in each paired comparison, based on their Z scores and p-values. Values highlighted in bold represent channels with statistically significant differences (*p* < 0.05) between the two steps or tasks. When possible, the values in the table are rounded to one or two decimal places.

## Comparison between holding conditions: power vs. precision

In this context, the generic terms “power grip” and “precision grip” will be employed to describe the actions associated with holding a hammerstone and holding a flake, respectively. It is important to note that while we use these terms interchangeably for simplification, it is not intended to suggest that holding a hammerstone or a flake is equivalent to any generic power grip and precision grip. The exact grips associated with a hammerstone and a flake are tailored to the distinct morphology and purpose of the tool (refer to Methods for a description of the grips used in the experiment).

The results of the Wilcoxon signed-rank test are presented in Supplementary Table S1. Statistically significant differences in beta power are revealed for channels FC1 (*p *= 0.03; Z=-2.14), CP1 (*p* = 0.03; Z=-2.24), O2 (*p* = 0.02; Z= -2.27), P8 (*p *= 0.02; Z=-2.27) and Cz (*p *= 0.04; Z=-2.11). These channels are located at contralateral frontocentral and centroparietal areas, ipsilateral occipital and temporal cortex, and central areas, respectively.

**Fig. 2:**
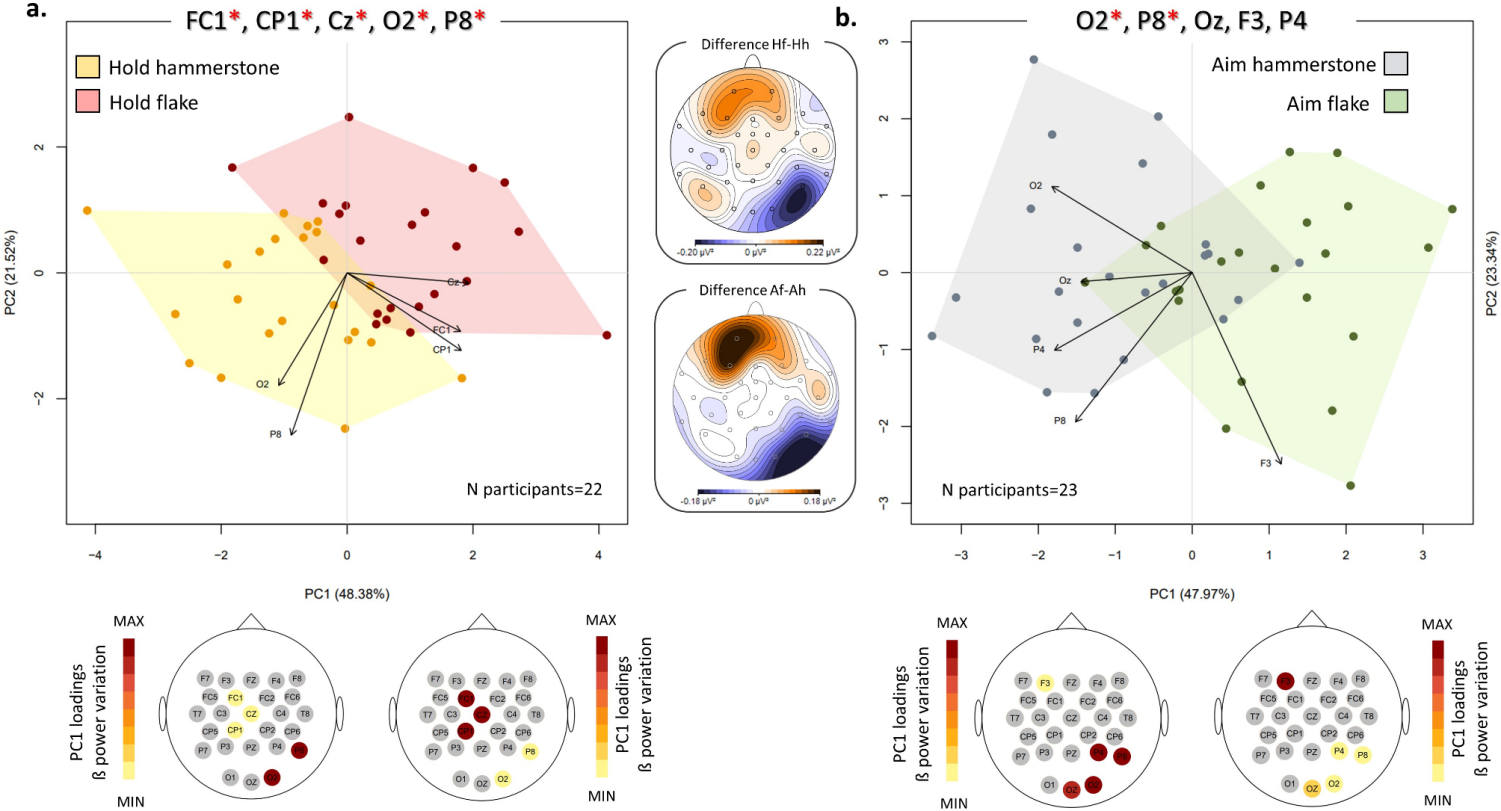
Paired comparisons of the two Hold conditions and the two Aim conditions. PCA plots illustrate the paired comparisons between Hold hammerstone (yellow) and Hold flake (red) (**a**), and between Aim hammerstone (grey) and Aim flake (green) (**b**). The plot depicts the first two components and the PCA analysis has been performed by using the mean power values of the 5 channels exhibiting the highest absolute Z scores as variables. Channels with significant differences between the two groups are marked with red asterisks. The maps below each PCA represent the loading values associated with each step/task, presented using a warm color palette that ranges from minimum to maximum values along PC1. The topographic maps between the two PCA plots represent the absolute beta power differences between Hold flake (“Hf”) and Hold hammerstone (“Hh”), and between Aim flake (“Af”) and Aim hammerstone (“Ah”).

These 5 channels were selected for the PCAs. The results are depicted in Fig. 2a (see also Supplementary Fig. S3 for grand-averaged mean beta topography). The first two principal components collectively account for about 70% of the variance (PC1 = 48.38%; PC2 = 21.52%). The observed variations between the precision grip (positive values of PC1) and the power grip (negative values of PC1) along PC1 are statistically significant (*p* = 0.001; Supplementary Table S2). Examination of PC loadings (refer to Supplementary Table S2 for loading values) suggests that this separation is predominantly driven by a relative increase in beta power in channels FC1, Cz, and CP1 for the precision grip. Along PC2, there is a proportional increase in beta power in channels O2 and P8 for the power grip, although these differences lack statistical significance.

We can further examine the differences between the two holding steps and the control task to provide a complete characterization of the overall beta power in each of the two conditions. The results of the Wilcoxon signed-rank test for the comparison of holding a flake and the control (Supplementary Table S3) revealed statistically significant differences in beta power for channels Fz (*p = 0.02*,* Z=-2.38*), F3 (*p = 0.002*,* Z=-3.15*), FC1 (*p = 0.02*,* Z=-2.44*), Cz (*p* = 0.003,* Z=-2.95*), CP1 (*p = 0.02*,* Z=-2.44*), and P4 (*p* = 0.02,* Z=-2.4*). These channels are situated in the contralateral frontal and frontocentral cortex, contralateral centroparietal and ipsilateral parietal areas, respectively.

The top five channels with the highest absolute Z scores were selected for PCA. The results are depicted in Fig. S4b (see also Supplementary Fig. S3 for grand-averaged mean beta topography). The first two PCs collectively account for about 80% of the total variance (PC1 = 61.56%; PC2 = 18.16%). Along PC1, a separation is observed between the precision grip and control conditions, with the precision grip characterized by a relative increase in beta power in all these channels compared to the control (*p* = 0.001; see Supplementary Table S4).

In contrast, when comparing holding a hammerstone and the control (see Supplementary Table S3), statistically significant differences in beta power are registered in channels P8 (*p* = 0.003,* Z=-2.96*), P4 (*p = 0.002*,* Z=-3.09*), Pz (*p* = 0.04,* Z=-2.05*), CP6 (*p* = 0.02,* Z=-2.44*), CP2 (*p* = 0.02,* Z=-2.31*), C4 (*p* = 0.0008,* Z=-3.35*), C3 (*p* = 0.04,* Z=-2.05*), FC1 (*p* = 0.02,* Z=-2.31*) and F3 (*p* = 0.04,* Z=-2.09*). These channels are located in the ipsilateral temporal, ipsilateral and central parietal cortex, ipsilateral and contralateral motor cortex, and contralateral frontal areas, respectively.

The top five channels with the highest absolute Z scores were selected for PCA. These include channels C4, CP6, P4, P8 and FC1. The first two PCs collectively account for about 83% of the total variance (PC1 = 65.44%; PC2 = 17.14%). Along PC1, a clear separation is observed between the power grip and control conditions (Fig. S4a), with the power grip characterized by a relative increase in beta power in all these channels compared to the control (*p* = 0.0001; see Supplementary Table S4).

## Comparison between aiming conditions

The results of the Wilcoxon signed-ranked test are presented in Supplementary Table S1. Statistical differences in beta power are found only for channels O2 (*p* = 0.005; Z=-2.78) and P8 (*p = 0.02; Z=-2.36*). Together with O2 and P8, channels Oz (*p* = 0.06; Z=-1.9), P4 (*p = 0.07; Z=-1.81*), and F3 (*p = 0.06; Z=-1.9*) were selected for PCA. These are located at the ipsilateral and central occipital cortex, ipsilateral parietal and temporal, and contralateral frontal areas.

The first two PCs explain 71.3% of the variance (PC1 = 47.97%; PC2 = 23.34%). The observed variations between aiming with a hammerstone (negative values of PC1) and aiming with a flake (positive values of PC1) along PC1 are statistically significant (*p = 0.0006;* Fig. 2b and Supplementary Table S2; see also Supplementary Fig. S3 for grand-averaged mean beta topography). Examination of PC loadings (refer to Supplementary Table S2 for loading values) suggests that this separation is predominantly influenced by a proportional increase in beta power in channels O2, Oz, P4, and P8 during hammerstone aiming, and in F3 during flake aiming.

In the paired comparison analysis between the two Aim conditions and the control, we identified statistically significant differences in beta power in 13 channels (Aim hammerstone versus Control) and 9 channels (Aim flake versus Control). These are listed in Supplementary Table S5 and include contralateral frontal and parietal areas for both flake and hammerstone, and ipsilateral parietal and temporal areas for hammerstone.

**Fig. 3:**
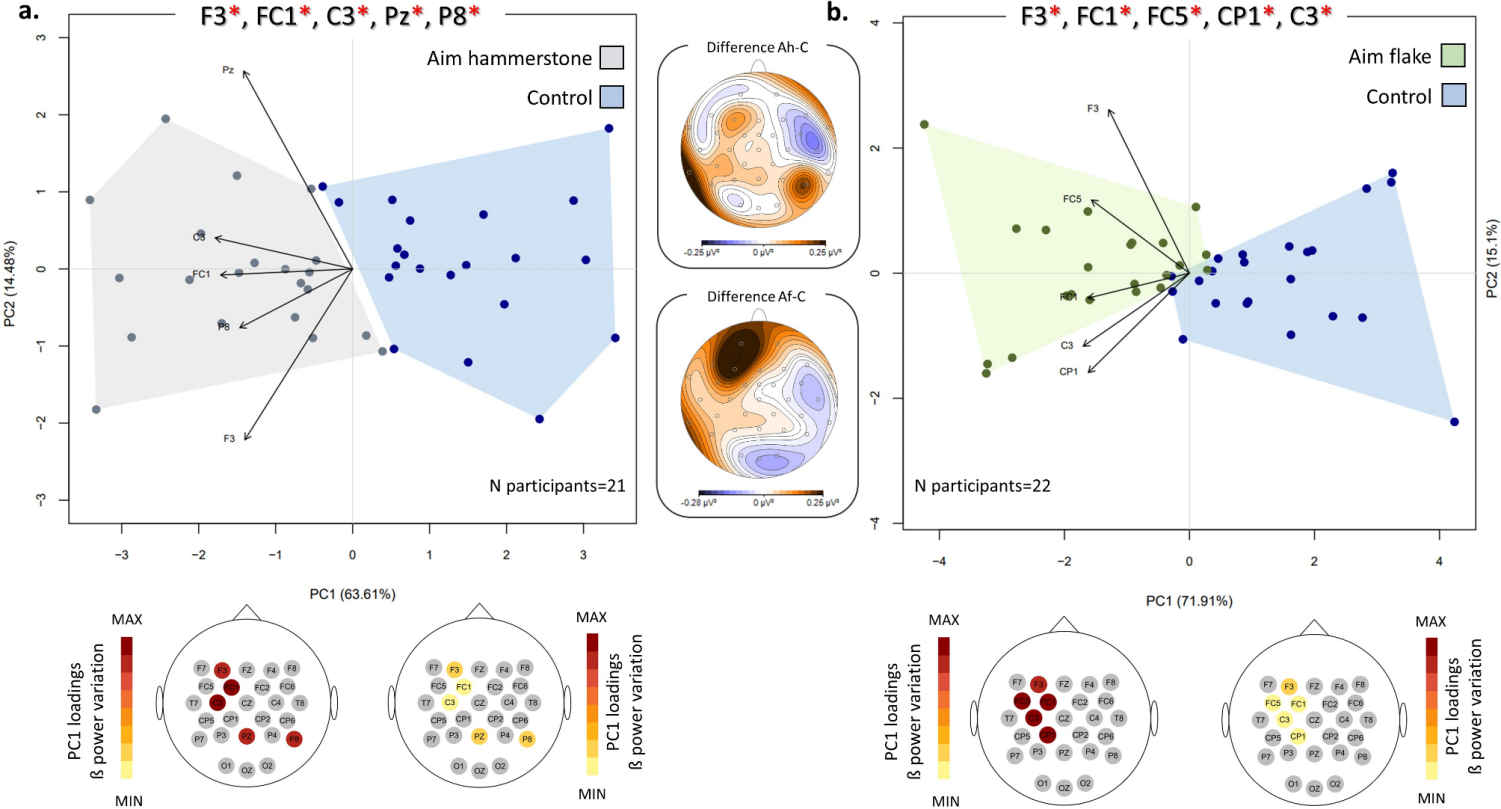
Paired comparisons of the two aiming conditions and the Control. PCA plots illustrate the paired comparisons between Aim hammerstone (grey) (**a**) or Aim flake (green) (**b**) and the Control (blue). The plot depicts the first two components and the PCA analysis has been performed by using the mean power values of the 5 channels exhibiting the highest absolute Z scores as variables. Channels with significant differences between the two groups are marked with red asterisks. The maps below each PCA represent the loading values associated with each step/task, presented using a warm color palette that ranges from minimum to maximum values along PC1. The topographic maps between the two PCA plots represent the absolute beta power differences between Aim hammerstone (“Ah”) and the Control (“C”), and between Aim flake (“Af”) and the Control (“C”).

In the first comparison (Aim hammerstone versus control), the channels selected for PCA are F3 (*p* = 0.0002; Z=-3.75), FC1 (*p* = 0.002; Z=-3.16), C3 (*p* = 0.001; Z=-3.27), Pz (*p = 0.008; Z=-2.64*) and P8 (*p* = 0.001; Z=-3.23). The first two principal components collectively explain 78% of the total variance, with PC1 accounting for 63.61% and PC2 for 14.48%. Along PC1, a separation is observed between the two groups (Fig. 3a), indicating a relative increase in beta power in all channels for the Aim hammerstone compared to the control (*p* = 4.768e-06; Supplementary Table S6).

In the second comparison (Aim flake versus control; Fig. 3b), the channels selected for PCA are F3 (*p* = 9.78E-05; Z=-3.9), FC1 (*p = 0.001; Z=-3.24*), FC5 (*p* = 0.003; Z=-3.02), C3 (*p* = 0.0005; Z=-3.47) and CP1 (*p* = 0.001; Z=-3.25). The first two PCs collectively account for 87% of the total variance (PC1 = 71.91%; PC2 = 15.1%). Along the PC1 axis, a relative increase in beta power is observed in all channels during flake aiming compared to the control (*p = 1.192e-05*; Supplementary Table S6).

### Comparison between steps of the same task

**Fig. 4:**
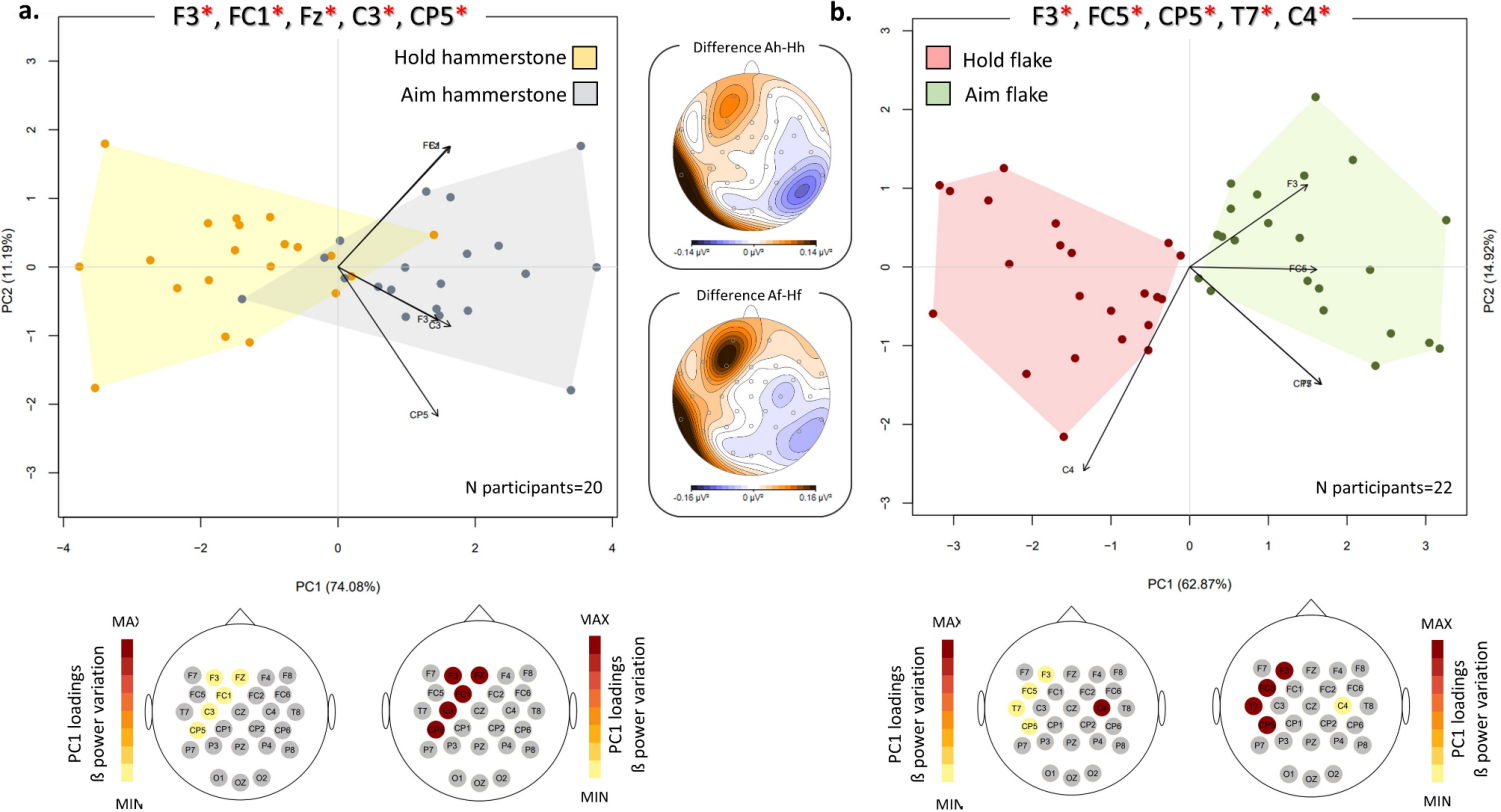
Paired comparison of the Aim and Hold within the nut-cracking task and flake-cutting task. PCA plots illustrate the paired comparisons between Hold hammerstone (yellow) and Aim hammerstone (grey) (**a**), and between Hold flake (red) and Aim flake (green) (**b**). The plot depicts the first two components and the PCA analysis has been performed by using the mean power values of the 5 channels exhibiting the highest absolute Z scores as variables. Channels with significant differences between the two groups are marked with red asterisks. The maps below each PCA represent the loading values associated with each step/task, presented using a warm color palette that ranges from minimum to maximum values along PC1. The topographic maps between the two PCA plots represent the absolute beta power differences between Aim hammerstone (“Ah”) and Hold hammerstone (“Hh”), and between Aim flake (“Af”) and Hold flake (“Hf”).

The results of the Wilcoxon signed-rank test for the comparison between “Hold” and “Aim” in the flake task are presented in Supplementary Table S7. Statistical differences in beta power are found for 10 channels and include contralateral frontal, parietal, and temporal areas, as well as ipsilateral frontal and parietal areas. The channels selected for PCA are F3 (*p = 0.0009; Z=-3.31*), FC5 (*p = 0.0004; Z=-3.54*), CP5 (*p* = 0.002; Z=-3.05), C4 (*p = 0.0002; Z=-3.67*), and T7 (*p* = 0.0002; Z=-3.7). The PCA plot is shown in Fig. 4b. The first two principal components collectively account for 77.8% of the variance (PC1 = 62.87%; PC2 = 14.92%). The observed variations between the Hold (negative values of PC1) and the Aim (positive values of PC1) along PC1 are statistically significant (*p* = 4.768e-07; see Supplementary Table S8). Examination of PC loadings (refer to Supplementary Table S8 for loading values) indicates that this differentiation primarily stems from a proportional increase in beta power in F3, FC5, CP5, and T7 during the aiming action.

**Fig. 5:**
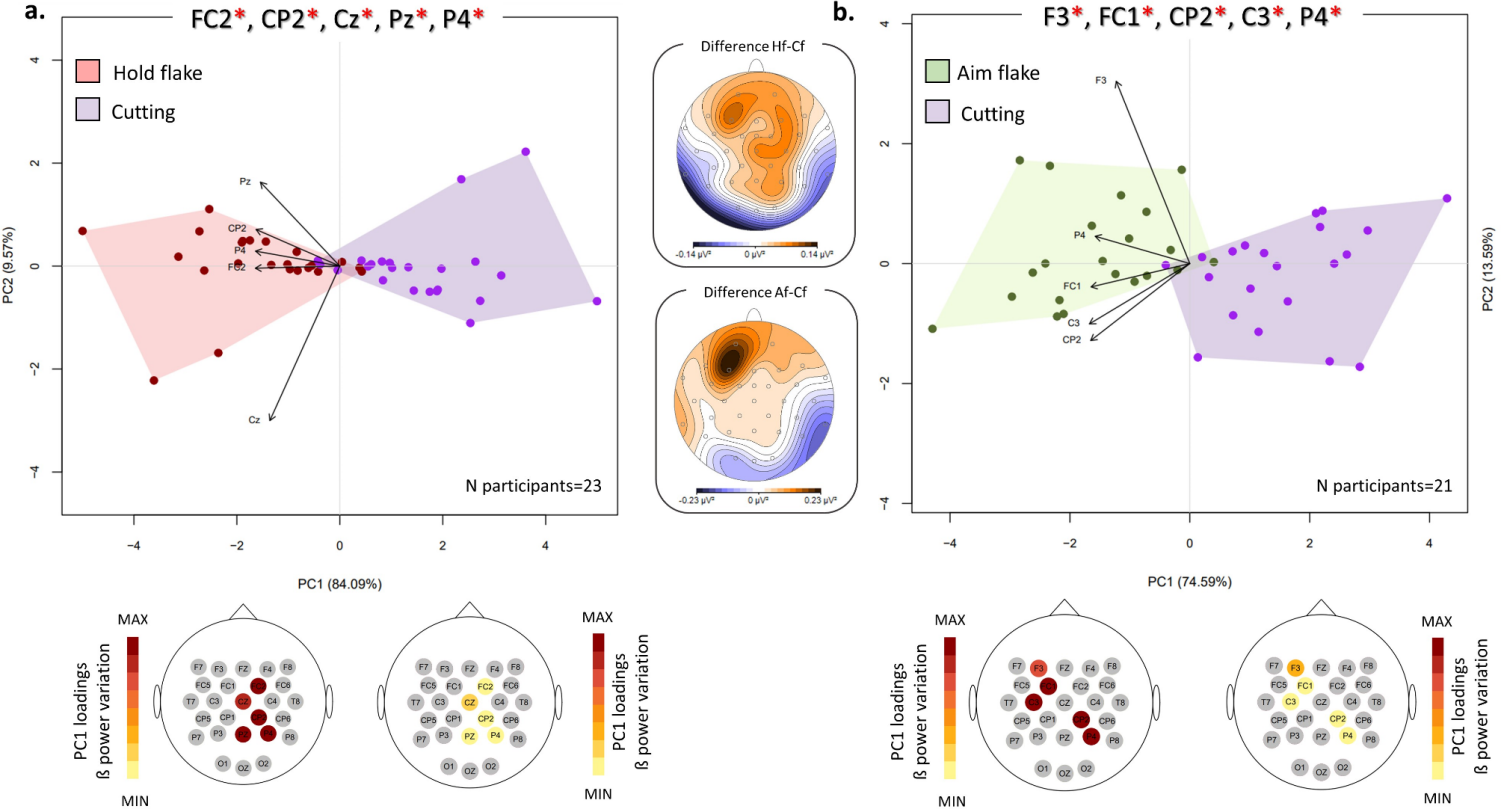
Paired comparison of the Aim/Hold and the Cutting step within the flake cutting task. PCA plots illustrate the paired comparisons between Hold flake (red) and flake Cutting (purple) (**a**), and between Aim flake (green) and flake Cutting (purple) (**b**). The plot depicts the first two components and the PCA analysis has been performed by using the mean power values of the 5 channels exhibiting the highest absolute Z scores as variables. Channels with significant differences between the two groups are marked with red asterisks. The maps below each PCA represent the loading values associated with each step/task, presented using a warm color palette that ranges from minimum to maximum values along PC1. The topographic maps between the two PCA plots represent the absolute beta power differences between Hold flake (“Hf”) and flake Cutting (“Cf”), and between Aim flake (“Af”) and flake Cutting (“Cf”).

Supplementary Table S7 presents the results of the Wilcoxon signed-rank test comparing Aim and Cutting in flake use. Statistically significant differences in beta power are observed for the majority of the channels (19 channels). Channels F3 (*p* = 9,*91E-05; Z=-3.89*), FC1 (*p* = 0.0003; Z=-3.58), CP2 (*p* = 0.0002; Z=-3.68), C3 (*p = 0.0005; Z=-3.51*), and P4 (*p = 0.0003; Z=-3.62*), located at the contralateral frontal and motor cortex, as well as ipsilateral centroparietal areas, were selected for PCA. The corresponding PCA plot is reported in Fig. 5b (see also Supplementary Fig. S3 for grand-averaged mean beta topography). The first two principal components explain 88.3% of the variance (PC1 = 74.59%; PC2 = 13.59%). Along PC1, a distinct separation is observed between the two conditions, with the aiming step characterized by a relatively increased beta power in all these channels compared to the cutting step (*p = 6.676e-06*; see Supplementary Table S8).

Similarly, in the comparison between flake Hold and Cutting, significant differences in beta power are observed in 13 channels (Supplementary Table S7). The 5 channels chosen for PCA exhibit a proportional increase in beta power during the holding phase (Fig. 5a). The separation along PC1 scores is statistically significant (*p* = 4.53e-06, refer to Supplementary Table S8), with channels FC2 (*p = 6*,35E-05, Z=-3.99), CP2 (*p = 4.30E-05*, Z=-4.09) and Cz (*p* = 0.0001,* Z=-3.88*) situated at ipsilateral frontoparietal and centroparietal areas, channel Pz (*p = 0.0003*, Z=-3.67) and P4 (*p = 0.0003*,* Z = 3.64*) at central and ipsilateral parietal areas.

Significant differences between flake Cutting and the Control are found only in channels C4 (*p* = 0.01,* Z=-2.57*) and FC2 (*p = 0.02*,* Z=-2.44*) (see Supplementary Fig. S5, Tables S9 and S10).

The findings from comparing the Hold and Aim steps in the hammerstone task are similar to those observed in the flake task. In this instance, statistical differences in beta power are found in 11 channels (Supplementary Table S11) and include contralateral frontal and parietal areas, as well as central and ipsilateral centroparietal areas. The channels selected for PCA are F3 (*p* = 0.0002; Z=-3.79), FC1 (*p* = 0.002; Z=-3.08), Fz (*p* = 0.001; Z=-3.19), C3 (*p* = 0.005; Z=-2.82), and CP5 (*p* = 0.005; Z=-2.82). Along PC1, a distinct separation is observed between the two conditions, with the aiming step characterized by a relatively increased beta power in all these channels compared to the holding condition (*p* = 0.0001; see Supplementary Table S12 and Fig. 4a).

## Discussion

The primary objective of this study was to investigate the cognitive demands associated with early hominin stone tool use within a real-time experimental framework, allowing for the comparison of cognitive processes across various stages of tool use. Our findings reveal distinct patterns of beta power across the different tasks (i.e., hammerstone nut-cracking and flake-cutting) and stages (i.e., holding, aiming, and flake-cutting).

We first hypothesized (1) that the fronto-parietal regions would be prominently involved during the execution of both stone tool use tasks. To test this hypothesis, we compared brain activity during hammerstone nut-cracking and flake-cutting against a control task. The control task involved a simple motor action without any tool manipulation, specifically chosen to primarily activate the motor cortex^69^. This comparison allowed us to isolate and examine the specific cognitive processes and brain regions involved in tool use, beyond basic motor functions. Our results indeed show that the fronto-parietal areas are more engaged in both tasks compared to the control. This increased engagement is evident across all stages of tool use, with the exception of the Execute stage: the results of the cutting step will be addressed in detail later in the discussion, while the nut-cracking stage could not be analyzed due to artifact contamination, as discussed in the Methods section.

The channels that appear recurrent in our analysis, showing significant differences in the beta band compared to the control, are channels F3 (all 4 comparisons), FC1 (all 4 comparisons), C3 (3 comparisons and borderline value in the flake-cutting task), CP1 (3 comparisons), and Pz (3 comparisons and borderline value in flake-cutting task). The increased beta power in these areas suggests heightened neural activity associated with the cognitive and motor demands of tool use. These regions are part of the broader tool-use network, which is involved in various aspects of planning, executing, and controlling tool-related actions^31,35,48^.

Our second hypothesis (2) posited that the precise cutting task would show a greater engagement of frontoparietal areas compared to the nut-cracking task. When examining the holding stages relative to the control, we observed distinct patterns of beta power activation. In the hold flake stage, there was a notable increase in beta power in the contralateral frontal and parietal lobe, as well as in one channel of the ipsilateral parietal lobe, indicating heightened engagement of this region. Conversely, the hold hammerstone stage exhibited a less extensive increase in beta power in the frontal lobe, but a notable increase in the ipsilateral motor, parietal, and posterior temporal lobe. When directly comparing the two holding stages, we further noted that the flake task showed an increase in beta power in the frontal and centroparietal channels, while the hammerstone task exhibited increased beta power in the ipsilateral posterior temporal and occipital cortex channels. Previous studies have identified distinct patterns of neural activity between power and precision grips, highlighting a stronger fronto-parietal interaction for precision grips^70,71^. These studies suggest that the late stages of precise motor grasps may require finer motor control, evidenced by activity over the medial pre-frontal cortex and bilateral motor and parietal regions as the moment of grasping onset approaches. While our results partially align with these studies, they also reveal a unique aspect: an increased activation in the ipsilateral parietal and temporo-occipital cortex during the power grip, which contrasts with the predominantly contralateral activity reported for power grips in previous research cited above.

This discrepancy might be explained by the nature of the task and the forces involved. Some studies have demonstrated a correlation between grip force and neural activation, suggesting a force-dependent modulation^72^. It is plausible that the nut-cracking task, which requires a power grip, involves higher force exertion, thereby increasing ipsilateral activity. Moreover, it is essential to consider that our study used EEG to specifically analyze beta power, while the aforementioned studies employed different imaging techniques and focused on overall neural activation. It is worth noting that our tasks involved functional and goal-directed tool use, which likely influenced the type of grasp and the associated neural activity. Additionally, the aiming and execution phases of our tasks involved bimanual activities, introducing a layer of complexity not typically present in previous fMRI studies, which predominantly utilized unimanual tasks. Bimanual tasks are likely to engage distinct neurocognitive processes due to the coordinated involvement of both hands. For example, during the aiming step, the non-dominant hand provides critical proprioceptive information, which supports the dominant hand in the subsequent execution phase. This proprioceptive feedback is integrated with visual input to optimize motor planning and coordination, potentially leading to more extensive neural recruitment (including involvement of ipsilateral regions) than is typically observed in unimanual tasks. In this context, the results of the nut-cracking task, particularly the increased engagement of ipsilateral areas during aiming, can be understood as requiring substantial gross motor coordination between both hands, where force and stabilization are key.

When comparing the aiming step to the control, we observed significant involvement of the fronto-parietal cortex, with a particularly strong activation in the frontal cortex. This pattern was evident both when comparing the aiming stages to the control and when comparing the aiming stages to the holding stages, regardless of the task (hypothesis 3). The heightened engagement of the frontal cortex during the aiming stage underscores its critical role in the planning and execution of precise motor actions^49,50,73^, such as positioning the flake or hammerstone before cutting or striking. Notably, when comparing the two holding stages with each other, no significant differences were found in this region, further highlighting the distinct cognitive demands of the aiming stage. The consistent involvement of the frontal cortex across both tasks during the aiming step suggests that this region is crucial for the anticipatory and preparatory processes required for effective tool use.

Surprisingly, while increased beta power over frontal and parietal regions was observed during the holding and aiming steps, the cutting (execution) stage did not show this increase. When comparing the cutting stage with the holding and aiming, an increase in beta power is evident for the holding and aiming steps. One possible explanation for this discrepancy is a decreased beta power during cutting due to induced event-related desynchronization (ERD), a well-known phenomenon of movement execution, mainly involving sensorimotor areas (e.g.,^74,75^). In our study, this step involved continuous movement, which may have resulted in a sustained beta ERD.

However, another plausible explanation might be that the critical cognitive processes required for successful cutting may have occurred earlier, during the holding and aiming stages, rather than being predominantly reflected in the cutting stage itself. During the holding and aiming stages, participants likely experienced heightened planning and decision-making demands, as they had to carefully position the flake and prepare for the cutting action. In contrast, the actual cutting stage may have been more automated, relying on previously well-established motor programs.

Another possibility is a design or analysis flaw specific to the cutting stage, although this seems highly unlikely given the consistent methodology applied across all stages. Additionally, it is possible that our focus on the beta band may have overlooked significant activity in other frequency bands, such as alpha, which is also known to be associated with different aspects of cognitive and motor processes (e.g.,^76,77^). Future research could benefit from a broader analysis across multiple frequency bands to provide an even deeper understanding of the cognitive demands of each stage.

According to one of the most fundamental assumptions surrounding hominin biocultural evolution, human-like tools, particularly those associated with fine precision tasks such as cutting, emerged concurrently with the genus *Homo* (and/or other early tool-using hominins) and were linked to encephalization and the fronto-parietal network (e.g.,^1,34^). Our study provides original empirical evidence to directly support this crucial connection by demonstrating distinct patterns of brain activation during different modes and stages of early hominin tool use. The involvement of the frontoparietal regions, particularly during the aiming stage of tool use, underscores the critical role of these brain areas in the planning and execution of motor actions^48,49,73^. The observed differences in beta power between the flake-cutting and nut-cracking tasks further emphasize the specialized neural mechanisms underlying precision tasks.

The fact that nut-cracking, a behavior that is also observed in extant non-human primates, does not elicit the same relative level of frontoparietal engagement falls in line with the hypothesis that an increased frontoparietal network may have been evolutionarily advantageous during early hominin evolution, benefitting types of tool use that only appear to be observed in hominins (i.e., cutting). Moreover, considering that the intentional production of a tool requires a clear understanding of its proper function and characteristics^33^, our findings may suggest that tool use was likely an essential precursor to the development of tool-making techniques (e.g., knapping). By emphasizing tool use as a foundational skill, we can better appreciate how early hominins evolved cognitively in response to the demands of their environment, gradually acquiring a better understanding of the shape of tools required for each necessary task and eventually conceiving how such tool forms can be produced using the available raw materials (especially when not available in nature).

Several experimental studies have shown that the fronto-parietal network plays a central role in both tool use and production (e.g.,^28^ and see in the review^31^). In fact, it has been demonstrated that active practice of Paleolithic toolmaking skills induces structural remodeling in the same brain regions that support tool use^78^, reflecting this study’s findings on the role of the fronto-parietal regions. Moreover, consistent with findings from stone tool production research (e.g.,^1,28^), our study on stone tool use also found that sensorimotor and cognitive demands generally tend to vary among different tasks. In particular, previous neuroimaging studies have shown that both Oldowan and Acheulean toolmaking engage the parietal cortex and the left ventral premotor cortex^1,28^. However, Acheulean toolmaking, in particular, shows significantly stronger activation in the frontal cortex, especially in the right hemisphere, reflecting the increased demands for effective hierarchical action organization^1^, which has also been linked to greater communicative capacity^79,80^. On this basis, future experimental research into the cognitive underpinnings of handaxe use, and how it compares to Oldowan flake use and/or stone tool production, may help achieve a more holistic understanding of early hominin cognition.

While our study provides original and valuable insights into the cognitive demands of early stone tool use, several limitations should be considered. Firstly, the EEG method, though effective in capturing real-time brain activity, has limited spatial resolution, making it challenging to pinpoint neural sources of activation more precisely. This constraint might have likely affected our ability to fully differentiate the contributions of closely situated brain regions within the fronto-parietal cortex. Future research could overcome this limitation by combining EEG with other neuroimaging techniques, such as functional near-infrared spectroscopy (fNIRS) or magnetoencephalography (MEG), which offer more precise spatial localization of brain activity (e.g.,^81^). Another future possibility would be to increase the number of electrodes, both to enhance spatial resolution and to allow for a more detailed examination of connectivity patterns. However, for this experiment, we chose to select this specific number of EEG electrodes due to experimental time constraints (considering the several demanding tasks to be performed and the recommendations of our university’s Ethics Committee), equipment availability, and to minimize the effort required from participants, thereby avoiding participant fatigue commonly observed in longer experiments. This decision was also based on ethical considerations and the approval received for the study. It should be highlighted that, regardless of the relatively small number of electrodes, we still found consistent, significant, and meaningful differences across tasks and steps, as well as relative to our control condition (also see^82^).

Secondly, artifact contamination in the nut-cracking (execution) stage data may have limited our ability to analyze this critical phase comprehensively, potentially obscuring additional findings. We plan to use different software packages in future studies to mitigate these artifacts and enable a thorough analysis. Additionally, further analyses may be used to elucidate the observed reduction in beta power during the cutting stage compared to the control and other task stages. Understanding this phenomenon will help to further confirm the cognitive processes involved.

Although our sample size of 23 participants is larger than that used in the vast majority of neuroimaging studies in neuroarchaeology and comparable to that of most EEG studies overall, its statistical power remains relatively low for hypothesis testing (e.g., Wilcoxon signed-rank tests), particularly in one of our analyses, which included only 18 individuals (see Fig. S4a). Even though we acknowledge the practical challenges involved in recruiting large numbers of participants for such experimental studies, we strongly recommend that future research prioritize increasing sample sizes to enhance the statistical power of their analyses.

Finally, while the controlled experimental setting and the techniques used here were necessary for isolating specific cognitive processes, they may arguably not fully replicate the naturalistic conditions under which early hominins used tools. Nonetheless, we made extensive efforts to simulate realistic conditions within the constraints of our laboratory environment and the technique employed, as well as to recruit a diverse participant group. Nevertheless, despite the diverse backgrounds of the 23 participants in our study, this sample cannot possibly represent the entire variability of humans or the characteristics of extinct species. Altogether, although our experimental design cannot possibly exactly replicate the activities of early hominins, it closely approximates their tool-use behaviors to the degree that this is feasible, providing valuable insights into the cognitive processes involved.

## Conclusions

This study put forth an original experimental and interdisciplinary approach to explore the cognitive demands associated with two fundamental modes of early hominin stone tool use: forceful nut-cracking and precise flake cutting. Its findings revealed distinct brain activity patterns throughout these activities, with the precise flake cutting task showing greater beta activation in the fronto-parietal regions of the brain. In both tasks, this signal was consistently more distinct in the aiming (planning) stage of the action. Overall, these results highlight the importance of these brain regions for planning and executing crucial tool-using precision tasks, confirming their decisive role in advanced motor control within the context of human-like early stone tool use. The observed distinction between nut-cracking (also observed in non-human primates) and cutting (exclusively associated with hominin contexts) provides original empirical support that the widely discussed modifications in these specific brain regions in hominins may have substantially benefited the early steps of biocultural evolution. Future research should build on these insights to further explore the intricate relationship between cognitive development and tool use.

## Electronic supplementary material

Below is the link to the electronic supplementary material.

Supplementary Material 1

## Data Availability

The datasets generated and analyzed during the current study are available from the corresponding author upon reasonable request.
