# Supplementary material for: Exploring the cognitive underpinnings of early hominin stone tool use through an experimental EEG approach
Source: Sci Rep. 2024 Nov 19;14:26936. doi: 10.1038/s41598-024-77452-0 (PMC11576949; doi:10.1038/s41598-024-77452-0)
Supplement: Supplementary file 1 — Supplementary Material 1 [file 41598_2024_77452_MOESM1_ESM.pdf]

## Exploring the cognitive underpinnings of early hominin stone tool use through an experimental EEG approach

## Supplementary Information

| EEG channels | Hold vs Hold | | Aim vs Aim | |
|---|---|---|---|---|
| | **Z scores** | **p.value** | **Z scores** | **p.value** |
| Fz | -1.266160359 | 0.205455676 | -0.076037383 | 0.939389357 |
| F3 | -1.720679463 | 0.085309005 | -1.900934577 | 0.057310582 |
| F7 | -0.03246565 | 0.974100709 | -0.167282243 | 0.867147975 |
| FC5 | -1.720679463 | 0.085309005 | -1.353465419 | 0.17590699 |
| FC1 | -2.142732916 | **0.032134551** | -0.076037383 | 0.939389357 |
| C3 | -0.162328251 | 0.871047368 | -0.410601869 | 0.681364493 |
| T7 | -0.681778655 | 0.495378923 | -0.410601869 | 0.681364493 |
| CP5 | -1.168763409 | 0.242498967 | -0.684336448 | 0.493762737 |
| CP1 | -2.240129866 | **0.025082493** | -0.106452336 | 0.915223471 |
| Pz | -1.460954261 | 0.144027994 | -0.927656074 | 0.353586001 |
| P3 | -0.194793901 | 0.845554297 | -0.89724112 | 0.369590267 |
| P7 | -0.389587803 | 0.696841373 | -0.380186915 | 0.703806671 |
| O1 | -0.941503857 | 0.346446714 | -1.110145793 | 0.266936207 |
| Oz | -1.785610763 | 0.074162303 | -1.900934577 | 0.057310582 |
| O2 | -2.272595517 | **0.023050566** | -2.782968221 | **0.005386408** |
| P4 | -1.201229059 | 0.22966236 | -1.809689717 | 0.070343918 |
| P8 | -2.272595517 | **0.023050566** | -2.357158876 | **0.018415366** |
| CP6 | -1.720679463 | 0.085309005 | -1.140560746 | 0.25405276 |
| CP2 | -1.298626009 | 0.194072307 | -0.684336448 | 0.493762737 |
| Cz | -2.110267265 | **0.034835342** | -0.288942056 | 0.77262572 |
| C4 | -0.324656502 | 0.745441059 | -1.353465419 | 0.17590699 |
| T8 | -0.129862601 | 0.896675134 | -0.015207477 | 0.987866657 |
| FC6 | -0.649313005 | 0.516136082 | -0.410601869 | 0.681364493 |
| FC2 | -1.493419911 | 0.135327304 | -0.349771962 | 0.726509842 |
| F4 | -1.233694709 | 0.217316686 | -0.836411214 | 0.402923606 |
| F8 | -1.071366458 | 0.284004689 | -0.258527102 | 0.796000135 |

**Table S1.** Wilcoxon signed-rank test results for the paired comparison of the two Hold conditions (i.e., Hold hammerstone vs Hold flake) and the two Aim conditions (i.e., Aim hammerstone vs Aim flake). Channels showing significant differences ($p<0.05$) and the highest absolute Z scores were selected for PCA. Significative p-values are highlighted in bold.

| Hold vs Hold | | | | | | | | |
|---|---|---|---|---|---|---|---|---|
| | **Eigenvalues** | **% Variance** | **Wilcoxon test PC scores** | **O2 loading** | **P8 loading** | **CP1 loading** | **FC1 loading** | **Cz loading** |
| PC1 | 155.53515 | 48.382366 | **p= 0.001262** | -0.310 | -0.253 | 0.518 | 0.515 | 0.553 |
| PC2 | 103.71934 | 21.515405 | p= 0.1129 | -0.511 | -0.736 | -0.352 | -0.266 | <-0.1 |
| PC3 | 87.86988 | 15.442231 | p= 0.8987 | 0.784 | -0.542 | <0.1 | -0.140 | 0.258 |
| PC4 | 64.36509 | 8.285730 | p= 1 | 0.167 | -0.161 | -0.472 | 0.801 | -0.285 |
| PC5 | 56.45471 | 6.374268 | p= 0.406 | <-0.1 | 0.271 | -0.617 | <-0.1 | 0.738 |
| Aim vs Aim | | | | | | | | |
| | **Eigenvalues** | **% Variance** | **Wilcoxon test PC scores** | **O2 loading** | **P8 loading** | **F3 loading** | **Oz loading** | **P4 loading** |
| PC1 | 154.87567 | 47.972947 | **p= 0.00055** | -0.521 | -0.433 | 0.330 | -0.413 | -0.511 |
| PC2 | 108.01624 | 23.335017 | p= 0.8229 | 0.320 | -0.555 | -0.711 | <-0.1 | -0.289 |
| PC3 | 87.62891 | 15.357651 | p= 0.8462 | 0.302 | <-0.1 | <0.1 | -0.840 | 0.446 |
| PC4 | 64.38193 | 8.290066 | p= 0.2467 | 0.392 | -0.619 | 0.555 | 0.327 | 0.220 |
| PC5 | 50.22110 | 5.044318 | p= 0.5009 | 0.618 | 0.343 | 0.275 | -0.128 | -0.639 |

**Table S2.** Principal Component Analysis statistics and loadings values for the paired comparison of the two Hold conditions (i.e., Hold hammerstone vs Hold flake) and the two Aim conditions (i.e., Aim hammerstone vs Aim flake). Significative p-values ($p<0.05$) are highlighted in bold.

| EEG channels | Hold flake vs Control | | Hold hammerstone vs Control | |
|---|---|---|---|---|
| | **Z scores** | **p.value** | **Z scores** | **p.value** |
| Fz | -2.369992467 | **0.017788448** | -1.916215648 | 0.055337652 |
| F3 | -3.149168073 | **0.00163736** | -2.09041707 | **0.036580351** |
| F7 | -0.389587803 | 0.696841373 | -0.566154623 | 0.571288674 |
| FC5 | -1.298626009 | 0.194072307 | -1.132309246 | 0.257504444 |
| FC1 | -2.434923768 | **0.014894927** | -2.308168848 | **0.020989747** |
| C3 | -1.915473364 | 0.055432163 | -2.046866715 | **0.040671173** |
| T7 | -0.259725202 | 0.795075751 | -0.522604268 | 0.60124967 |
| CP5 | -0.714244305 | 0.475076125 | -1.611363158 | 0.107100592 |
| CP1 | -2.434923768 | **0.014894927** | -1.262960313 | 0.206603443 |
| Pz | -1.785610763 | 0.074162303 | -2.046866715 | **0.040671173** |
| P3 | -1.168763409 | 0.242498967 | -1.69846387 | 0.089420246 |
| P7 | -0.973969507 | 0.33007168 | -1.480712091 | 0.138683311 |
| O1 | -0.811641256 | 0.416997511 | -0.087100711 | 0.93059146 |
| Oz | -1.038900808 | 0.298850871 | -1.785564581 | 0.074169786 |
| O2 | -1.103832108 | 0.269665974 | -1.437161736 | 0.150672043 |
| P4 | -2.402458118 | **0.016285299** | -3.09207525 | **0.001987625** |
| P8 | -1.038900808 | 0.298850871 | -2.961424183 | **0.003062199** |
| CP6 | -0.714244305 | 0.475076125 | -2.438819915 | **0.014735309** |
| CP2 | -1.947939014 | 0.051422261 | -2.308168848 | **0.020989747** |
| Cz | -2.954374172 | **0.003133039** | -0.827456757 | 0.407978222 |
| C4 | -1.818076413 | 0.069052454 | -3.353377384 | **0.000798318** |
| T8 | -0.649313005 | 0.516136082 | -0.914557468 | 0.360423995 |
| FC6 | -0.584381704 | 0.558963533 | -0.69680569 | 0.485924405 |
| FC2 | -1.655748162 | 0.097772829 | -0.827456757 | 0.407978222 |
| F4 | -0.454519103 | 0.649455245 | -0.130651067 | 0.896051343 |
| F8 | -0.324656502 | 0.745441059 | -0.174201423 | 0.861707168 |

**Table S3.** Wilcoxon signed-rank test results for the paired comparison of the Hold conditions (i.e., Hold flake or Hold hammerstone) and the Control. Channels showing significant differences and the highest absolute Z scores were selected for PCA. Significative p-values ($p<0.05$) are highlighted in bold.

| Hold flake vs Control | | | | | | | | |
|---|---|---|---|---|---|---|---|---|
| | **Eigenvalues** | **% Variance** | **Wilcoxon test PC scores** | **F3 loading** | **Cz loading** | **FC1 loading** | **CP1 loading** | **P4 loading** |
| PC1 | 175.44861 | 61.564428 | **p= 0.0013** | -0.305 | -0.489 | -0.488 | -0.465 | -0.462 |
| PC2 | 95.29641 | 18.162811 | p= 0.4826 | 0.808 | 0.190 | <0.1 | -0.295 | -0.473 |
| PC3 | 74.08586 | 10.977428 | p= 0.7745 | -0.431 | 0.417 | 0.554 | -0.545 | -0.191 |
| PC4 | 52.76854 | 5.569037 | p= 0.5661 | -0.253 | 0.601 | -0.395 | 0.429 | -0.485 |
| PC5 | 43.16420 | 3.726297 | p= 0.5879 | <0.1 | 0.436 | 0.547 | -0.464 | 0.540 |
| Hold hammerstone vs Control | | | | | | | | |
| | **Eigenvalues** | **% Variance** | **Wilcoxon test PC scores** | **P4 loading** | **P8 loading** | **C4 loading** | **CP6 loading** | **FC1 loading** |
| PC1 | 180.88813 | 65.441030 | **p= 0.00011** | -0.492 | -0.388 | -0.481 | -0.425 | -0.442 |
| PC2 | 92.57548 | 17.140438 | p= 0.2645 | 0.241 | 0.702 | -0.278 | -0.609 | <0.1 |
| PC3 | 68.06741 | 9.266346 | p= 0.8317 | 0.182 | 0.173 | 0.408 | <0.1 | -0.874 |
| PC4 | 46.76560 | 4.374043 | p= 0.9323 | 0.749 | -0.563 | <-0.1 | -0.348 | <0.1 |
| PC5 | 43.46346 | 3.778144 | p= 0.6705 | -0.325 | <-0.1 | 0.724 | -0.566 | 0.201 |

**Table S4.** Principal Component Analysis statistics and loadings values for the paired comparison of the Hold conditions (i.e., Hold flake or Hold hammerstone) and the Control. Significative p-values (p<0.05) are highlighted in bold.

| EEG channels | Aim flake vs Control | | Aim hammerstone vs Control | |
|---|---|---|---|---|
| | **Z scores** | **p.value** | **Z scores** | **p.value** |
| Fz | -2.369992467 | **0.017788448** | -2.467793148 | **0.013594885** |
| F3 | -3.895878028 | **9.78E-05** | -3.753826197 | **0.000174156** |
| F7 | -1.493419911 | 0.135327304 | -1.564094249 | 0.117795436 |
| FC5 | -3.019305472 | **0.002533549** | -2.050701348 | **0.04029604** |
| FC1 | -3.246565024 | **0.001168068** | -3.162946147 | **0.001561812** |
| C3 | -3.473824575 | **0.000513096** | -3.267219097 | **0.001086096** |
| T7 | -1.39602296 | 0.162707581 | -0.2085459 | 0.834802748 |
| CP5 | -2.499855068 | **0.012424412** | -2.606823748 | **0.009138639** |
| CP1 | -3.246565024 | **0.001168068** | -2.502550798 | **0.012330193** |
| Pz | -2.142732916 | **0.032134551** | -2.641581398 | **0.008251998** |
| P3 | -2.142732916 | **0.032134551** | -2.328762548 | **0.019871648** |
| P7 | -0.973969507 | 0.33007168 | -1.737882498 | 0.08223152 |
| O1 | -1.038900808 | 0.298850871 | -0.31281885 | 0.754418294 |
| Oz | -1.103832108 | 0.269665974 | -1.251275399 | 0.210834018 |
| O2 | -1.39602296 | 0.162707581 | -1.112244799 | 0.266032911 |
| P4 | -1.33109166 | 0.183158853 | -2.606823748 | **0.009138639** |
| P8 | -0.486984754 | 0.626269136 | -3.232461447 | **0.001227287** |
| CP6 | -0.357122153 | 0.721000361 | -1.216517749 | 0.223787754 |
| CP2 | -1.558351211 | 0.119150015 | -1.772640148 | 0.076288355 |
| Cz | -1.850542064 | 0.064235461 | -2.259247248 | **0.023868009** |
| C4 | -0.389587803 | 0.696841373 | -0.556122399 | 0.578127183 |
| T8 | -1.33109166 | 0.183158853 | -1.807397798 | 0.070700277 |
| FC6 | -1.493419911 | 0.135327304 | -2.050701348 | **0.04029604** |
| FC2 | -0.779175606 | 0.435876278 | -1.042729499 | 0.297073591 |
| F4 | -0.649313005 | 0.516136082 | -0.2780612 | 0.78096538 |
| F8 | -0.324656502 | 0.745441059 | -0.764668299 | 0.444469088 |

**Table S5.** Wilcoxon signed-rank test results for the paired comparison of the two Aim conditions (i.e., Aim hammerstone vs Aim flake) and the Control. Channels showing significant differences and the highest absolute Z scores were selected for PCA. Significative p-values ($p<0.05$) are highlighted in bold.

| Aim flake vs Control | | | | | | | | |
|---|---|---|---|---|---|---|---|---|
| | **Eigenvalues** | **% Variance** | **Wilcoxon test PC scores** | **F3 loading** | **C3 loading** | **FC1 loading** | **CP1 loading** | **FC5 loading** |
| PC1 | 189.61621 | 71.908615 | **p= 1.192e-05** | -0.369 | -0.485 | -0.463 | -0.462 | -0.448 |
| PC2 | 86.90401 | 15.104615 | p= 0.6102 | 0.747 | -0.334 | -0.114 | -0.454 | 0.334 |
| PC3 | 54.76944 | 5.999384 | p= 0.799 | 0.507 | 0.246 | -0.490 | 0.367 | -0.555 |
| PC4 | 52.48087 | 5.508483 | p= 0.8237 | -0.223 | 0.183 | -0.729 | 0.129 | 0.607 |
| PC5 | 27.19286 | 1.478903 | p= 0.6102 | <-0.1 | 0.748 | <-0.1 | -0.655 | -0.107 |
| Aim hammerstone vs Control | | | | | | | | |
| | **Eigenvalues** | **% Variance** | **Wilcoxon test PC scores** | **F3 loading** | **C3 loading** | **P8 loading** | **FC1 loading** | **Pz loading** |
| PC1 | 178.34221 | 63.611884 | **p= 4.768e-06** | -0.400 | -0.510 | -0.419 | -0.490 | -0.405 |
| PC2 | 85.08942 | 14.480420 | p= 0.412 | -0.633 | 0.115 | -0.218 | <-0.1 | 0.733 |
| PC3 | 75.02560 | 11.257682 | p= 0.7335 | 0.524 | 0.190 | -0.805 | <-0.1 | 0.181 |
| PC4 | 57.30157 | 6.566940 | p= 0.7593 | -0.322 | <0.1 | -0.358 | 0.787 | -0.375 |
| PC5 | 45.18337 | 4.083075 | p= 0.5168 | -0.245 | 0.826 | <-0.1 | -0.363 | -0.354 |

**Table S6.** Principal Component Analysis statistics and loadings values for the paired comparison of the two Aim conditions (i.e., Aim hammerstone vs Aim flake) and the Control. Significative p-values ($p<0.05$) are highlighted in bold.

| EEG channels | Hold vs Aim (flake) | | Aim vs Cutting (flake) | | Hold vs Cutting (flake) | |
|---|---|---|---|---|---|---|
| | **Z scores** | **p.value** | **Z scores** | **p.value** | **Z scores** | **p.value** |
| Fz | -0.714244305 | 0.475076125 | -2.815369647 | **0.004872116** | -3.087117753 | **0.002021075** |
| F3 | -3.311496324 | **0.000927985** | -3.892856796 | **9.91E-05** | -2.570063548 | **0.010167986** |
| F7 | -1.947939014 | 0.051422261 | -2.850127297 | **0.004370173** | -2.478818689 | **0.013181829** |
| FC5 | -3.538755876 | **0.000402017** | -3.197703797 | **0.001385265** | -1.779274764 | 0.075194726 |
| FC1 | -1.655748162 | 0.097772829 | -3.580037947 | **0.000343544** | -3.0567028 | **0.00223786** |
| C3 | -2.889442871 | **0.003859251** | -3.510522647 | **0.000447227** | -2.661308408 | **0.007783763** |
| T7 | -3.701084127 | **0.00021468** | -2.502550798 | **0.012330193** | -1.292635512 | 0.196137151 |
| CP5 | -3.051771122 | **0.002274955** | -1.529336599 | 0.126181021 | -1.596785045 | 0.110313631 |
| CP1 | -1.071366458 | 0.284004689 | -3.371492047 | **0.000747622** | -3.178362613 | **0.001481094** |
| Pz | -0.876572556 | 0.380718851 | -3.267219097 | **0.001086096** | -3.665001865 | **0.000247337** |
| P3 | -1.39602296 | 0.162707581 | -2.780611997 | **0.005425654** | -2.357158876 | 0.018415366 |
| P7 | -1.720679463 | 0.085309005 | -0.834183599 | 0.404177532 | -1.110145793 | 0.266936207 |
| O1 | -0.194793901 | 0.845554297 | -0.695152999 | 0.486959424 | -0.410601869 | 0.681364493 |
| Oz | -0.616847355 | 0.537335414 | -0.868941249 | 0.384879266 | 0 | 1 |
| O2 | -0.941503857 | 0.346446714 | -0.10427295 | 0.916952744 | -0.866826167 | 0.386037262 |
| P4 | -2.207664216 | **0.027267689** | -3.614795597 | **0.000300585** | -3.634586911 | **0.000278426** |
| P8 | -1.460954261 | 0.144027994 | -2.120216648 | **0.033987779** | -0.076037383 | 0.939389357 |
| CP6 | -2.045335965 | **0.040821747** | -1.633609549 | 0.102340862 | -0.197697196 | 0.843281984 |
| CP2 | -2.402458118 | **0.016285299** | -3.684310897 | **0.000229322** | -4.09081121 | **4.30E-05** |
| Cz | -1.168763409 | 0.242498967 | -3.371492047 | **0.000747622** | -3.877906537 | **0.000105359** |
| C4 | -3.668618477 | **0.000243865** | -2.259247248 | **0.023868009** | -3.482512145 | **0.000496733** |
| T8 | -1.006435157 | 0.314206289 | -2.015943698 | **0.043805863** | -1.049315887 | 0.294032757 |
| FC6 | -1.103832108 | 0.269665974 | -0.38233415 | 0.702213528 | -1.383880372 | 0.166395088 |
| FC2 | -1.818076413 | 0.069052454 | -3.475764997 | **0.000509398** | -3.99956635 | **6.35E-05** |
| F4 | -0.162328251 | 0.871047368 | -2.050701348 | **0.04029604** | -3.300022426 | **0.000966771** |
| F8 | -2.369992467 | **0.017788448** | -2.224489598 | **0.026115522** | -1.748859811 | 0.080315255 |

**Table S7.** Wilcoxon signed-rank test results for the paired comparison between steps of the flake task. Channels showing significant differences and the highest absolute Z scores were selected for PCA. Significative p-values ($p<0.05$) are highlighted in bold.

| Hold vs Aim (flake) | | | | | | | | |
|---|---|---|---|---|---|---|---|---|
| | **Eigenvalues** | **% Variance** | **Wilcoxon test PC scores** | **T7 loading** | **C4 loading** | **FC5 loading** | **F3 loading** | **CP5 loading** |
| PC1 | 177.29290 | 62.865548 | **p= 4.768e-07** | 0.478 | -0.384 | 0.461 | 0.428 | 0.477 |
| PC2 | 86.36731 | 14.918624 | p= 0.2756 | -0.426 | -0.740 | <-0.1 | 0.298 | -0.426 |
| PC3 | 71.43501 | 10.205921 | p= 0.5661 | <0.1 | 0.309 | -0.514 | 0.796 | <-0.1 |
| PC4 | 61.40582 | 7.541349 | p= 0.3053 | 0.277 | -0.456 | -0.723 | -0.299 | 0.321 |
| PC5 | 47.26816 | 4.468558 | p= 0.4434 | 0.712 | <-0.1 | <0.1 | <-0.1 | -0.697 |
| Aim vs Cutting (flake) | | | | | | | | |
| | **Eigenvalues** | **% Variance** | **Wilcoxon test PC scores** | **F3 loading** | **CP2 loading** | **P4 loading** | **FC1 loading** | **C3 loading** |
| PC1 | 193.12350 | 74.593372 | **p= 6.676e-06** | -0.351 | -0.472 | -0.451 | -0.471 | -0.478 |
| PC2 | 82.42882 | 13.589021 | p= 0.6333 | 0.868 | -0.367 | 0.130 | -0.111 | -0.287 |
| PC3 | 56.42860 | 6.368373 | p= 0.8917 | -0.231 | 0.104 | 0.821 | -0.415 | -0.298 |
| PC4 | 43.73089 | 3.824781 | p= 0.179 | 0.244 | 0.448 | -0.242 | -0.750 | 0.346 |
| PC5 | 28.49959 | 1.624453 | p= 0.7335 | 0.103 | 0.657 | -0.217 | 0.176 | -0.693 |
| Hold vs Cutting (flake) | | | | | | | | |
| | **Eigenvalues** | **% Variance** | **Wilcoxon test PC scores** | **CP2 loading** | **FC2 loading** | **Cz loading** | **Pz loading** | **P4 loading** |
| PC1 | 205.05205 | 84.092690 | **p= 4.53e-06** | -0.465 | -0.469 | -0.388 | -0.441 | -0.468 |
| PC2 | 69.18818 | 9.574007 | p= 0.2861 | 0.203 | <-0.1 | -0.857 | 0.466 | <0.1 |
| PC3 | 41.97633 | 3.524024 | p= 0.5399 | 0.508 | 0.525 | -0.224 | -0.564 | -0.314 |
| PC4 | 29.65052 | 1.758307 | p= 0.6869 | -0.123 | <-0.1 | -0.225 | -0.509 | 0.821 |
| PC5 | 22.92348 | 1.050972 | p= 0.7998 | 0.685 | -0.709 | 0.116 | -0.110 | <0.1 |

**Table S8.** Principal Component Analysis statistics and loadings values for the paired comparison between steps of the flake task. Significative p-values ($p<0.05$) are highlighted in bold.

| EEG channels | **Z scores** | **p.value** |
|---|---|---|
| Fz | -1.071366458 | 0.284004689 |
| F3 | -1.136297758 | 0.255831969 |
| F7 | -0.876572556 | 0.380718851 |
| FC5 | -0.292190852 | 0.770140706 |
| FC1 | -0.681778655 | 0.495378923 |
| C3 | -0.973969507 | 0.33007168 |
| T7 | -0.486984754 | 0.626269136 |
| CP5 | -1.168763409 | 0.242498967 |
| CP1 | -0.0649313 | 0.948228699 |
| Pz | -0.486984754 | 0.626269136 |
| P3 | -0.227259552 | 0.820221923 |
| P7 | -0.129862601 | 0.896675134 |
| O1 | -1.655748162 | 0.097772829 |
| Oz | -0.584381704 | 0.558963533 |
| O2 | -1.298626009 | 0.194072307 |
| P4 | -0.616847355 | 0.537335414 |
| P8 | -0.941503857 | 0.346446714 |
| CP6 | -0.844106906 | 0.398609669 |
| CP2 | -1.233694709 | 0.217316686 |
| Cz | -1.071366458 | 0.284004689 |
| C4 | -2.564786369 | **0.010323935** |
| T8 | -0.486984754 | 0.626269136 |
| FC6 | -1.785610763 | 0.074162303 |
| FC2 | -2.434923768 | **0.014894927** |
| F4 | -1.460954261 | 0.144027994 |
| F8 | -1.103832108 | 0.269665974 |

**Table S9.** Wilcoxon signed-rank test results for the paired comparison of the Cutting step and the Control. Channels showing significant differences and the highest absolute Z scores were selected for PCA. Significative p-values ($p<0.05$) are highlighted in bold.

| | Eigenvalues | % Variance | Wilcoxon test PC scores | C4 loading | FC2 loading | FC6 loading | O1 loading | F4 loading |
|---|---|---|---|---|---|---|---|---|
| PC1 | 154.98957 | 48.043532 | **p= 0.0067** | -0.603 | -0.574 | -0.476 | -0.274 | <0.1 |
| PC2 | 112.11900 | 25.141340 | p= 0.2479 | 0.192 | 0.103 | -0.465 | 0.379 | 0.770 |
| PC3 | 94.34792 | 17.803059 | p= 0.6789 | <0.1 | 0.266 | 0.151 | -0.836 | 0.451 |
| PC4 | 56.82924 | 6.459125 | p= 0.7502 | <-0.1 | -0.541 | 0.701 | 0.178 | 0.424 |
| PC5 | 35.72775 | 2.552944 | p= 0.8736 | 0.769 | -0.544 | -0.209 | -0.225 | -0.135 |

**Table S10.** Principal Component Analysis statistics and loadings values for the paired comparison of Cutting and the Control. Significative p-values ($p<0.05$) are highlighted in bold.

| EEG channels | **Z scores** | **p.value** |
|---|---|---|
| Fz | -3.191943255 | **0.001413191** |
| F3 | -3.789265969 | **0.000151093** |
| F7 | -1.773301808 | 0.07617871 |
| FC5 | -2.445289862 | **0.014473577** |
| FC1 | -3.079945246 | **0.002070387** |
| C3 | -2.818616558 | **0.00482311** |
| T7 | -1.698636469 | 0.089387701 |
| CP5 | -2.818616558 | **0.00482311** |
| CP1 | -2.370624523 | **0.017758061** |
| Pz | -0.653321719 | 0.513548894 |
| P3 | -1.922632487 | 0.054526221 |
| P7 | -2.221293844 | **0.026331066** |
| O1 | -0.57865638 | 0.562821054 |
| Oz | -0.50399104 | 0.614267673 |
| O2 | -0.317327692 | 0.750994967 |
| P4 | -1.54930579 | 0.121308228 |
| P8 | -1.62397113 | 0.10438197 |
| CP6 | -2.445289862 | **0.014473577** |
| CP2 | -0.802652397 | 0.422175674 |
| Cz | -2.370624523 | **0.017758061** |
| C4 | -2.146628505 | **0.031822869** |
| T8 | -1.138646424 | 0.254850657 |
| FC6 | -0.877317737 | 0.380314081 |
| FC2 | -1.026648415 | 0.304586048 |
| F4 | -0.242662353 | 0.808266974 |
| F8 | -0.279995022 | 0.779481324 |

**Table S11.** Wilcoxon signed-rank test results for the paired comparison between steps of the hammerstone task (i.e., Hold vs Aim). Channels showing significant differences and the highest absolute Z scores were selected for PCA. Significative p-values ($p<0.05$) are highlighted in bold.

| | Eigenvalues | % Variance | Wilcoxon test PC scores | F3 loading | FC1 loading | Fz loading | C3 loading | CP5 loading |
|---|---|---|---|---|---|---|---|---|
| PC1 | 192.45602 | 74.078640 | **p= 0.000134** | 0.417 | 0.468 | 0.462 | 0.469 | 0.416 |
| PC2 | 74.79406 | 11.188304 | p= 0.4524 | -0.223 | 0.501 | 0.502 | -0.247 | -0.621 |
| PC3 | 65.97644 | 8.705783 | p= 0.4524 | 0.859 | -0.150 | <-0.1 | -0.100 | -0.469 |
| PC4 | 48.65791 | 4.735184 | p= 0.9273 | 0.166 | -0.173 | 0.410 | -0.765 | 0.436 |
| PC5 | 25.41741 | 1.292089 | p= 0.498 | 0.107 | 0.691 | -0.597 | -0.352 | 0.176 |

**Table S12.** Principal Component Analysis statistics and loadings values for the paired comparison between steps of the hammerstone task (i.e., Hold vs Aim). Significative p-values ($p<0.05$) are highlighted in bold.

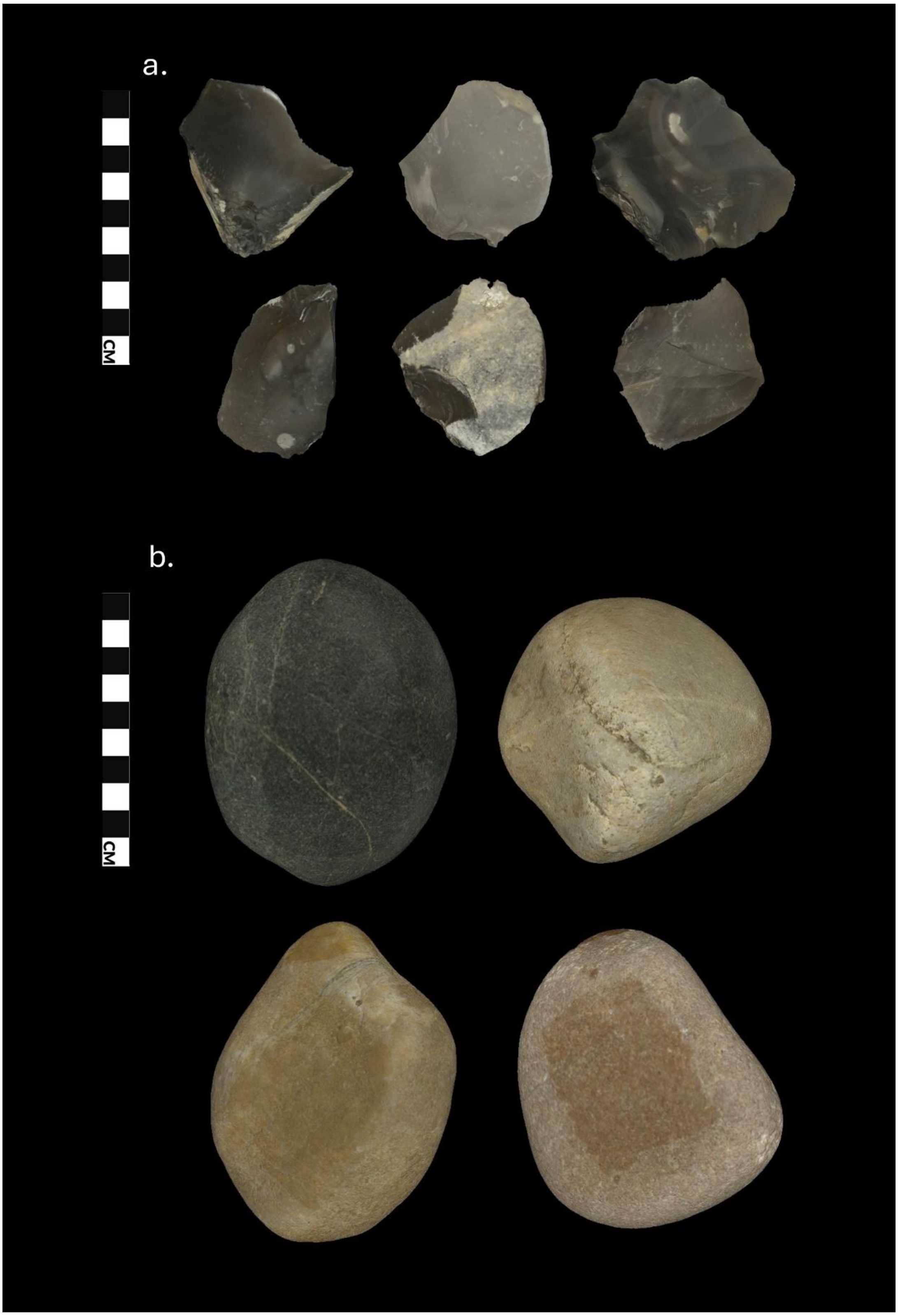


**Figure S1.** Selection of Oldowan flake (a) and hammerstone (b) replicas used in the study. The tools in the figure were 3D scanned using Artec SpaceSpider (Artec Inc.), with a resolution of 50 microns.

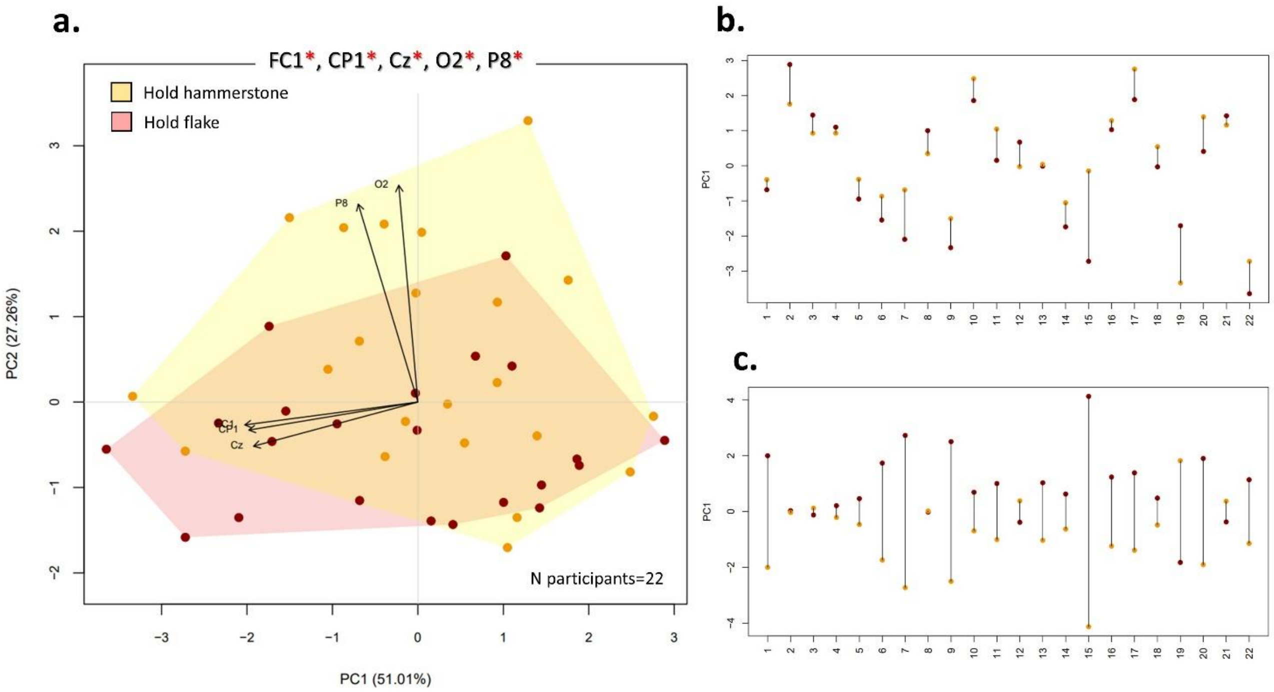


**Figure S2.** PCA plot illustrating the paired comparison between holding a hammerstone (yellow) and holding a stone flake (red) before mean adjustment (a). The plot depicts the first two components and PCA has been performed by using the mean power values of the 5 channels exhibiting the highest absolute Z scores as variables. Channels with significant differences between the two groups are marked with red asterisks. Drop-line graphs of PC1 scores (component with statistically significant variations between the two groups) before mean adjustment (b) and after mean adjustment (c). Despite an observed overlap in the PCA plot of the two groups, a discernible trend is evident in the drop-line graph (b). Importantly, this trend persists when comparing the two drop-line graphs. Refer to main text's Fig. 1 for the same PCA plot with mean adjustment and note the visual improvement.

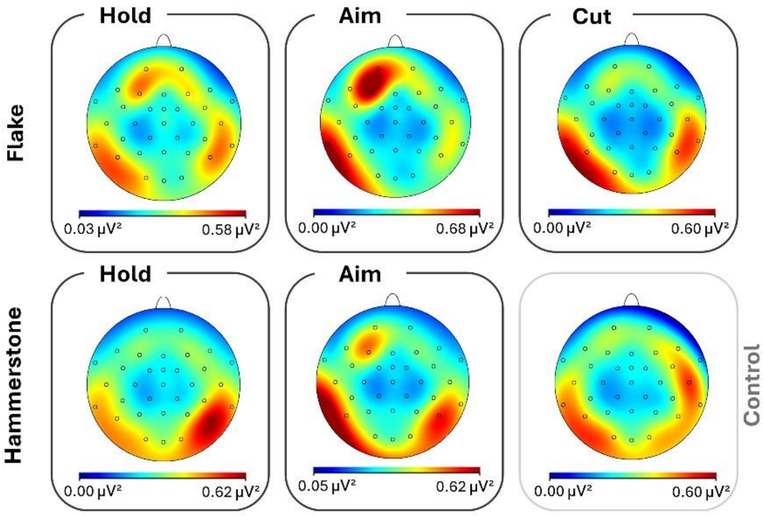


**Figure S3.** Topographic map representing scalp distribution of mean beta power (µV²) in each task step averaged over 23 participants. The red color represents higher values, and the blue color represents lower values.

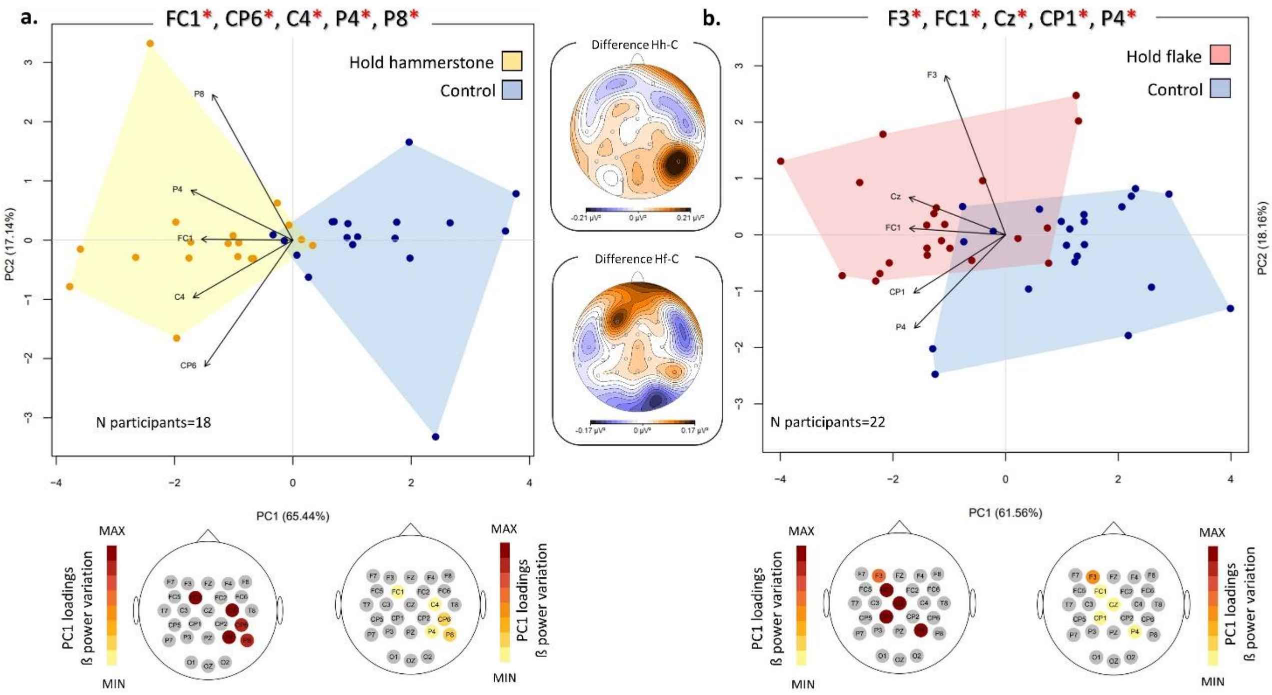


**Figure S4.** Paired comparisons of the two holding conditions and the Control. PCA plots illustrate the paired comparisons between Hold hammerstone (yellow) (a) or Hold flake (red) (b) and the Control (blue). The plot depicts the first two components and the PCA analysis has been performed by using the mean power values of the 5 channels exhibiting the highest absolute Z scores as variables. Channels with significant differences between the two groups are marked with red asterisks. The maps below each PCA represent the loading values associated with each step/task, presented using a warm color palette that ranges from minimum to maximum values along PC1. The topographic maps between the two PCA plots represent the absolute beta power differences between Hold hammerstone (“Hh”) and the Control (“C”), and between Hold flake (“Hf”) and the Control (“C”).

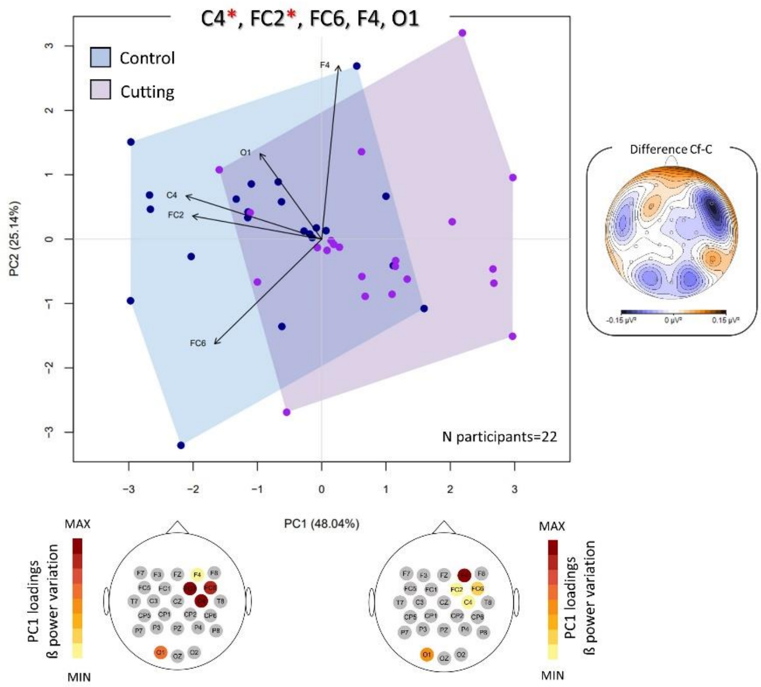


**Figure S5**. Paired comparison of the flake Cutting and the Control. PCA plot illustrates the paired comparison between flake Cutting (purple) and the Control (blue). The plot depicts the first two components and the PCA analysis has been performed by using the mean power values of the 5 channels exhibiting the highest absolute Z scores as variables. Channels with significant differences between the two groups are marked with red asterisks. The maps below the PCA represent the loading values associated with each step/task, presented using a warm color palette that ranges from minimum to maximum values along PC1. The topographic map on the right side of the plot represents the absolute beta power differences between flake Cutting ("Cf") and the Control ("C").
